# Prognostic value of immune cells in the tumor microenvironment of early-stage lung cancer: a meta-analysis

**DOI:** 10.18632/oncotarget.27392

**Published:** 2019-12-24

**Authors:** Stephanie Tuminello, Rajwanth Veluswamy, Wil Lieberman-Cribbin, Sacha Gnjatic, Francesca Petralia, Pei Wang, Raja Flores, Emanuela Taioli

**Affiliations:** ^1^Institute for Translational Epidemiology and Department of Population Health Science and Policy, Icahn School of Medicine at Mount Sinai, New York, NY, USA; ^2^Department of Medicine, Hematology and Medical Oncology, Icahn School of Medicine at Mount Sinai, New York, NY, USA; ^3^Department of Oncological Sciences, Icahn School of Medicine at Mount Sinai, New York, NY, USA; ^4^Precision Immunology Institute, Icahn School of Medicine at Mount Sinai, New York, NY, USA; ^5^Department of Genetics and Genomic Science, Icahn School of Medicine at Mount Sinai, New York, NY, USA; ^6^Department of Thoracic Surgery, Icahn School of Medicine at Mount Sinai, New York, NY, USA; ^7^Tisch Cancer Institute, Icahn School of Medicine at Mount Sinai, New York, NY, USA

**Keywords:** NSCLC, immune contexture, tumor microenvironment, TILs

## Abstract

Background: Early-stage non-small cell lung cancer (NSCLC) patients carry significant risk of recurrence post-surgery. In-depth characterization of the immune tumor microenvironment (TME) can have prognostic value. This study aimed to evaluate the association of individual immune cell types in the TME with clinical outcomes in surgically resected, early-stage NSCLC.

Methods: We performed a systematic literature search of the National Library of Medicine database through November 2019, investigating predefined biomarkers (CD3+ T cells, CD4+ T helper cells, CD8+ cytotoxic T cells, CD20+ B cells, CD56+ & CD57+ Natural Killer (NK) cells, CD68+ Tissue Associated Macrophages (TAMS), FoxP3+ T regulatory cells, and Mast Cells (MC)), and their association with survival following PRISMA guidelines.

Results: Studies that adjusted for important clinical covariates (such as stage and age) showed that higher levels of CD8+ cytotoxic T cells were associated with improved OS (HR = 0.68; 95% CI, 0.50–0.93) and DFS (HR = 0.60; 95% CI, 0.41–0.87), while increased CD20+ B cells (HR = 0.16; 95% CI, 0.04–0.64) and CD 56/57+ NK cells (HR = 0.50; 95% CI, 0.26–0.95) were associated with improved OS; lung cancers with increased FoxP3+ T regulatory cells (HR = 2.22; 95% CI, 1.47–3.34) had worse OS.

Conclusions: Immune cell components of the TME have prognostic value in early-stage, surgically resected NSCLC, and may reveal which patients are more likely to need additional systemic treatment, including immunotherapy. Clinical covariates need to be considered when evaluating the prognostic value of immune cells in the TME.

## INTRODUCTION

Immune cells within the tumor microenvironment (TME) play an important role in the development, progression and outcomes of non-small cell lung cancer (NSCLC). Innate and adaptive immune cells are able to detect and eliminate malignant transformed cells through the process of immunosurveillance [1]. However, lung cancers that become clinically apparent acquire resistance mechanisms to escape the anti-tumor immune response [1–3]. The relative balance of antagonistic effector (i.e., CD8+ cytotoxic T cells) and regulatory (i.e., FoxP3+ T regulatory cells [Tregs]) immune cell subpopulations may tilt the TME to be either detrimental or supportive of tumorigenesis, and will have a profound impact on the tumor’s eventual destiny [4]. Therefore in-depth characterization of the immune cell composition of the TME is critical to understanding cancer outcomes and may help guide treatment decisions for lung cancer patients.

Resection of early stage NSCLC represents the best opportunity for meaningful long-term survival and cure. However, despite complete removal of all detectable disease, there remains a significant risk of lung cancer recurrence [5]. Predicting which patients are most likely to have recurrence following surgery is of great clinical importance. Patients within the same TNM stage exhibit wide variations in recurrence rates [5]. Efforts to improve post-surgical outcomes using adjuvant chemotherapy have only provided marginal benefit in a subset of patients, while exposing all patients to significant toxicity. New prognostic biomarkers based on immune cell signatures that predict survival outcomes of early stage NSCLC can help identify patients that are most likely to receive benefit from additional systemic treatment, including neo-adjuvant or adjuvant immunotherapies which are now being studied.

A number of retrospective studies have shown individual immune cells within the TME are associated with survival in various malignancies. In colorectal cancers, the type and density of tumor infiltrating lymphocytes (TILs) were found to be more powerful prognostic factors than standard anatomical staging criteria (i.e., TNM) [6]. While CD8+ TILs have been the most studied, mounting data is demonstrating many other immune cell types in the TME also play an important role in cancer outcomes [7]. However, findings from these studies are often not consistent and are limited by small sample size. Three previous meta-analyses evaluating the association between immune cells in lung cancer outcomes are also limited because they included studies on patients with both local and advanced disease and did not account for potentially important clinical confounders [8–10].

Here, we have conducted a meta-analysis of studies evaluating the association of individual immune cell types in the TME with clinical outcomes of surgically resected, early stage NSCLC in order to determine novel prognostic biomarkers for this subset of patients.

## MATERIALS AND METHODS

### Search strategy

We conducted a systematic literature search of the National Library of Medicine database to search for all original, retrospective observational studies reporting postoperative survival outcomes of surgically resected, stage I-III NSCLC patients according to immune cell biomarkers (measured by immunohistochemistry [IHC]). There were no date restrictions and the search was finalized in November 2019. Additionally, the cited references of each study (including published reviews and other meta-analyses) were reviewed and evaluated for eligibility. The specific immune cell types studied were selected based on prior literature showing their role in lung cancer prognosis and included: CD3+ T cells, CD4+ T helper cells, CD8+ cytotoxic T cells, CD20+ B cells, CD56+ & CD57+ Natural Killer (NK) cells, CD68+ Tissue Associated Macrophages (TAMS), FoxP3+ T regulatory cells, and Mast Cells (MC). For each of these biomarkers the search terms included: the relevant biomarker, “lung cancer”, and “survival”. A separate Medline search was performed for each biomarker.

### Selection criteria

Articles were first screened for relevancy within the scope of the project by independent review of titles and abstracts by two reviewers (ST and WLC). Articles that met qualification underwent further scrutiny through full text review to assure they met all the selection criteria. Disagreements in screening and selection were adjudicated by group consensus involving a third reviewer (ET). Studies were considered eligible for inclusion in this systematic review if they reported on: 1) human subjects, 2) stage I-III NSCLC patients who underwent surgical resection, 3) at least 10 patients, 4) survival data for at least one of the predefined biomarkers or provided sufficient data to estimate survival, and 5) follow-up of at least 4 years. Outcomes of interest were overall survival (OS) and disease-free survival (DFS), either as unadjusted estimates or after adjustment for clinical covariates.

### Data extraction

All relevant descriptive information was extracted from each study to create a standardized tabular summary, including author, year of publication, biomarker data, tumor histology, tumor stage, duration of follow-up, number of patients included, gender (when reported), smoking status (when reported), and hazard ratio [HR] and 95% confidence interval [CI]) for OS and/or DFS. When studies reported separate survival for immune cell biomarkers in the tumor compartment and the stromal compartment, tumor HR was preferentially chosen over the stromal HR as tumor infiltrating cells are likely more clinically relevant.

### Statistical methods

A meta-analysis was conducted using a linear mixed-effects model to determine the meta-estimate of the average effect, either OS or DFS [11]. The presence of heterogeneity across studies was tested with the Q and I^2^ statistics [12]. The results of the meta-analyses were graphically summarized using forest plots created with the metafor package in R Studio (version 3.2.2; R Foundation for Statistical Computing, Vienna, Austria) [13].

## RESULTS

**Figure 1:**
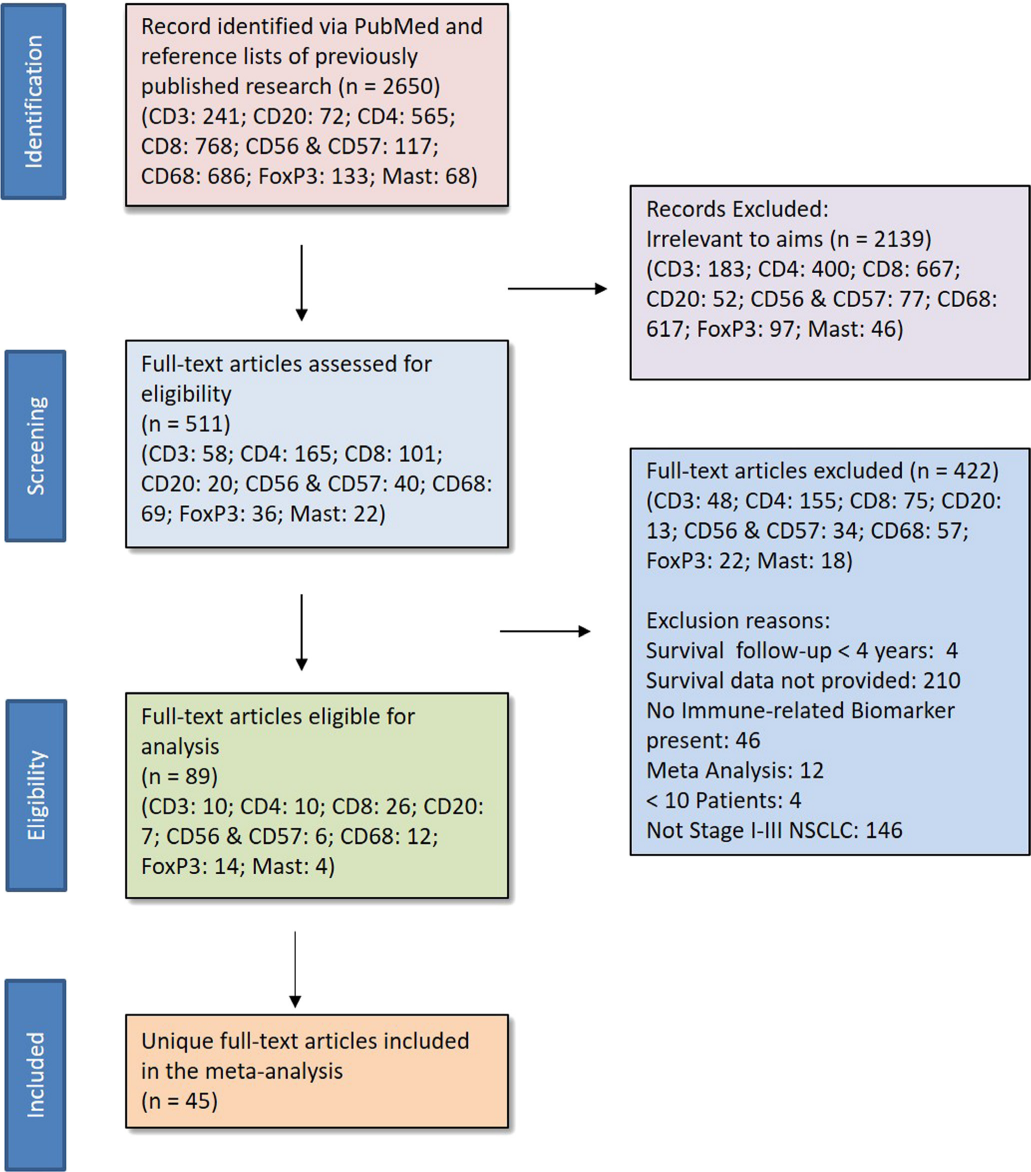
PRISMA. * Eligibility Criteria 1) human subjects, 2) stage I-III NSCLC patients who underwent surgical resection, 3) at least 10 patients, 4) survival data for at least one of the predefined biomarkers or provided sufficient data to estimate survival, and 5) follow-up of at least 4 years.

We identified and screened for relevance 2,650 studies; 2,139 of them were irrelevant to the aims of the study. The full text of the remaining 511 articles were reviewed for inclusion, and 45 unique studies were found to be eligible, accounting for 8,471 patients (see Figure 1 for Preferred Reporting Items for Systematic Reviews and Meta-analyses [PRISMA] guidelines). Reasons for ineligibility included having reported survival less than 4 years after surgical resection, inclusion of stage IV NSCLC cases, and not having reported a HR and 95% CI. Of the included studies, 22 reported survival measures for multiple biomarkers of interest, and 16 studies reported both unadjusted and adjusted HRs (Table 1).

**Table 1:** Characteristics of included studies

Study, Year	Biomarker	Location	Outcome	HR	Covariates in adjustment model	*n* of Patients	Male (%)	Stage (%)	Histology (%)	Smoking Status	Cutoff Value
Takanami, 2000 [25]	MCs	Tumor	OS	Adj	gender, T, N, differentiation, microvascular density	180	56	I: 55	ADC: 100	NR	MCD>21
								II: 14			
								III: 31			
Pelletier, 2001 [26]	CD20	Peritumor	OS	Adj	stage, histology, gender	113	60	I: 58	ADC: 50	Current: 92%	>10%
								II: 18	SCC: 42		
								III: 24	LCC: 3		
									Other: 5		
Kojima, 2002 [27]	CD68 MCs	Intratumor	OS	Unadj and Adj	VEGF, microvessel density	132	67	I: 100	ADC: 71	NR	Mean
									SCC: 29		
Villegas, 2002 [28]	CD57	Intratumor	OS	Unadj and Adj	stage, age, endoscopy localization	50	98	I: 82	SCC: 100	Current: 74%	Median
								II: 12		Former: 22%	
								III: 6		Never: 4%	
Pelosi, 2004 [29]	MCs	Tumor	OS & DFS	Unadj and Adj	age, diameter, pT, Ki-67 labelling index, HER-2, tumor grade, symptoms, blc-2, synaptophysin	201	91	I: 100	ADC: 44	NR	≥5%
									SCC: 56		
Kojima, 2005 [30]	CD68 MCs	Tumor		Unadj and Adj (adenocarcinoma only)	tumor VEGF, tumor VEGF-C, tumor VEGFR-3, microvessel density	129	48	I: 100	ADC: 100	Smoker: 40%	Staining score >3
										Non-smoker: 60%	
Petersen, 2006 [31]	CD3	Tumor	DFS	Unadj	n/a	64	53	I: 100	ADC: 46	Mean pack-year 51 ± 33	Score ≥2 (median)
									SCC: 34		
									Other: 19		
Kikuchi, 2007 [32]	CD8	Cancer-Nest	OS	Unadj	n/a	161	68	I: 59^*^	ADC: 52	Never: 24%	1-cell increment
	CD56							II/III/IV: 41	SCC: 42	Ever: 67%	
									Other: 6	Unknown: 9%	
Dieu-Nosjean, 2008 [33]	CD3	Tumor	OS & DFS	Unadj	n/a	74	81	I: 84	ADC: 62	Current: 91%	1.5 mean score
	CD20							II: 16	SCC: 38	Never: 9%	
Shimizu, 2010 [34]	FoxP3	Intratumor	DFS	Adj	nodal involvement, COX-2 expression	100	60	I: 68	ADC: 69	NR	≥3%
								II: 14	SCC: 31		
								III: 18			
da Costa Souza, 2012 [35]	CD4		OS	Adj	stage and histological type	65	69	I: 31	ADC: 58	Pack-years median 41(0–120)	CD4: ≥16.1%
	CD8							II: 51	SCC: 31		CD8: ≥1.8%
	CD68							III: 18	LCC: 11		CD68: ≥4.5%
Hanagiri, 2013 [36]	FoxP3	Regional Lymph Nodes	OS	Unadj and Adj	gender, pack-year index, T factor, N factor	158	65	I: 72	ADC: 59	NR	>0.5% PBL
								II: 11	SCC: 22		>1.1% RLNL
								III: 19	Other: 9		
Suzuki, 2013 [37]	FoxP3	Stroma	DFS	Unadj and Adj	gender, stage, lymphatic invasion, IL-12R, IL-7R	956 (478)	36	I: 100	ADC: 100	Current: 16%	Score ≥2
										Former: 69%	
										Never: 15%	
Feng, 2014 [38]	CD68	Islet and Stroma	OS & DFS	Adj	mediastinal down staging, islet/stromal machrophage ratio	28	54	III: 100	ADC: 70	Smoker: 39%	median
									SCC: 21	Never: 61%	
Germain, 2014 [39]	CD20	Tumor	OS	Unadj	n/a	74	81	I: 84	ADC: 62	Current: 91%	CD20: 0.0255 mm^2^/tumor IPF
								II: 16	SCC: 38	Never: 9%	
Hanagiri, 2014 [40]	FoxP3	Regional Lymph Nodes	OS	Unadj and Adj	gender, age, histology	131	57	I: 100	ADC: 76	NR	Relative expression > 0.06
									SCC: 16		
									Other: 8		
Lee, 2014 [41]	CD68		DFS	Adj	procedure, stage, CD68, FoxP3/CD3	151	40	I: 100	ADC: 77	Current: 7%	Score ≥2
									SCC: 23	Former: 72%	
										Never: 21%	
Djenidi, 2015 [42]	CD3	Total (tumor + stroma)	OS & DFS	Unadj	n/a	101	68	I: 100	ADC: 45	Ever: 89%	Continuous variables
	CD8								SCC: 42		
									Other: 13		
Hernandez-Prieto, 2015 [43]	CD3	Stroma	DFS	Unadj	n/a	84	86	I: 71	ADC: 48	Current: 43%	Moderate 10%
	CD4							II: 29	SCC: 46	Former: 51%	Strong 20%
	CD8								LCC: 2	Never: 6%	
	CD20								Other: 4		
	CD57										
Kadota, 2015 [44]	CD3	Tumor	OS	Unadj	n/a	331	60	I: 57	SCC: 100	≤90 pack-year: 81%	CD3: High ≥50
	CD4							II: 28		>90 pack-year: 19%	CD4: High ≥20
	CD8							III: 15			CD8: High ≥50
	CD20										CD20: High ≥20
	CD68										CD68: High ≥50
	FoxP3										FoxP3: High ≥20
	Neutrophil-(CD10)										CD10: High ≥10
Kim, 2015 [45]	CD8	Tumor	OS & DFS	Unadj	n/a	331	96	I: 40	SCC: 100	Smoker: 91%	Median
								II: 36		Never: 5%	
								III: 24			
Donnem, 2015 [46]	CD8	Stroma	OS & DFS	Adj	stage, differentiation, histology, age	797	64	I: 51	ADC: 48	NR	High density >50%
								II: 35	SCC: 45		
								III: 15	LCC: 7		
Li, 2015 [47]	CD68		OS & DFS	Adj	TNM, N stage, subcarinal lymph node, *n* number of nodal stations involved, number of involved nodes	159	69	I: 23	ADC: 26	Smoker: 31%	
								II: 36	SCC: 52	Never: 69%	
								III: 24			
O’Callaghan, 2015 [48]	CD3	TI/S	OS	Unadj and Adj	tumor size, stage, lymph node stage, WHO overall stage, positive resection margins, TI/S biomarker ratios	197 (186)	62	I: 55	ADC: 45	Current & Former: 94%	Median
	CD8							II: 24	SCC: 46	Never: 6%	
	FoxP3							III: 21	LCC: 3		
									Other: 6		
Paulsen, 2015 [49]	CD8	Tumor	OS	Unadj	n/a	536	68	I: 48	ADC: 38	Current: 33%	score of ≥2
								II: 36	SCC: 54	Former: 64%	
								III: 16		Never: 3%	
Tian, 2015 [50]	CD3	Tumor	OS	Adj	histology, pT, pN, pTNM stage, CD3, CD8, IL-2	129	71	I: 37	ADC: 37	Smoker: 37%	H-score 0–4 = low expression
	CD8							II: 22	SCC: 47	Never: 63%	H-score 5–12 = high expression
								III: 40	Other: 16		
Ameratunga, 2016 [51]	CD8	Stroma	OS & DFS	Unadj and Adj	age, sex, pneumonectomy status, nodal stage, histology, smoking status, CD8+, FOXP3, PD-L1	527 (509)	69	NR	ADC: 55	Heavy: 73%	Score of % of positively stained cells > 3
									SCC: 34	Light: 17%	
									Other: 11	Never: 7%	
										Unknown: 4%	
Kinoshita, 2016 [52]	CD4	Tumor	OS & DFS	Unadj and Adj (for CD20 only)	age, diameter, nodal metastasis, pleural invasion, FoxP3/CD4	218	65	I: 71	ADC: 72	Ever: 61%	CD4: > 251 cells/mm^2^
	CD8							II: 11	SCC: 18	Never: 39%	CD8: > 110 cells/mm^2^
	CD20							III: 18	Other: 10		FoxP3: > 163 cells/mm^2^
	FoxP3										
Parra, 2016 [53]	CD57	Peri and intra tumor	OS (CD57 only) & DFS	Adj	tumor stage, adjuvant therapy	254	55	I: 50	ADC: 57	Current: 45%	Median
	CD68							II: 30	SCC: 43	Former: 30%	
								III: 20		Never: 17%	
Teng, 2016 [54]	CD8	Tumor	OS & DFS	Unadj and Adj (for CD8 only)	high risk, FoxP3/CD8+TILS	126	67	I: 100	ADC: 45	NR	CD3: ≥30%
	FoxP3								SCC: 33		FoxP3: ≥45/HPF
									LCC: 22		
Uso, 2016 [55]	CD8	Tumor and Stroma	OS & DFS (CD8 only)	Adj	Tumoral CD8+ cells, Stromal FOXP3+ cells, FOXP3+ stroma/CD4+ tumor, FOXP3+ stroma/CD8+ tumor	122	85	I: 59	ADC: 42	Current: 48%	CD8: Median
	FoxP3							II: 21	SCC: 47	Former: 38%	FoxP3: >10%
								III: 20	Other: 11	Never: 14%	
Yang, 2016 [56]	CD4	Tumor and Stroma	OS & DFS	Adj	age, sex, stage, PD-L1 expression, Stromal CD4+ T cell, Stromal regulatory T cell, Epithelial CD8+ T cell	105	85	I: 100	SCC: 100	Smoker: 75%	CD4: ≥5% (tumor) & ≥25% (stroma)
	CD8									Non Smoker: 25%	CD8: ≥5% (tumor) & ≥50% (stroma)
	FoxP3										FoxP3: ≥20%
Yazdi, 2016 [57]	CD8	Stroma	OS	Unadj and Adj	stage, sex, HLA-E	197	50	I: 31	ADC: 100	NR	mean
								II: 38			
								III: 18			
Huang, 2017 [58]	CD8	Tumor	OS & DFS	Adj	age, sex, differentiation, histology, tumor, node, CD133, OCT-4, CD8, CD56, HLA I, PD-L1	172	65	NR	SCC: 42	Smoker: 55%	Median
	CD56								Other: 58	Non-Smoker: 45%	
Kinoshita, 2017 [59]	CD4	Tumor	OS & DFS	Unadj	n/a	164	5	I: 68	ADC: 100	Non-smoker: 100%	CD4: > 585 cells/mm^2^
	CD8							II, III: 32			CD8: > 900 cells/mm^2^
	CD20										CD20: > 1070 cells/mm^2^
	FoxP3										FoxP3: > 81 cells/mm^2^
Koh, 2017 [60]	CD8 [60]	Tumor	DFS	Adj	age, lymph node metastasis, CD103+ TILs, CD8+ TILs	378	95	I, II: 79	SCC: 100	Smoker: 90%	mean
								III: 21		Never: 5%	
Sepesi, 2017 [61]	CD3	Intratumor	OS	Unadj and Adj	age, histology, thoracotomy, adjuvant therapy, DLCO %, Zubrod score, Lobectomy, PD-L1 tumor H score, PD-L1 tumor % expression, PD-L1 macrophages H score, PD-L1 macrophages % expression, CD3, CD4, CD8, CD45RO, CD57, CD68, FoxP3, PD-1	113	49	I: 100	ADC: 70	Current and Former: 89%	CD3: ≥ 827.3
	CD4								SCC: 30	Never: 11%	CD4 ≥ 852.1
	CD8										CD8: ≥ 292.3
	CD57										CD57: ≥ 1408
	CD68										CD68: ≥ 515
	FoxP3										FoxP3: ≥ 460.3
Ye, 2017 [62]	CD8		OS	Unadj and Adj	gender, age, pathology grade, T, N, clinical stage	102	54	I: 20	ADC: 100	NR	final score>6
								II: 59			
								III: 21			
Barua, 2018 [63]	CD4	Tumor	OS	Unadj	n/a	120	43	I: 4	ADC: 60	Current & Former: 83%	G-cross signatures
	CD8							II: 11	SCC: 29	Never: 17%	
	CD68							III: 85	Other: 11		
	FoxP3										
Jackute, 2018 [64]	CD68	Total	OS	Unadj	n/a	80	80	I: 29	ADC: 48	Smoker: 83%	Median
								II: 33	SCC: 45	Non-Smoker: 17%	
								III: 39	Other: 7		
Mazzaschi, 2018 [65]	CD3		OS & DFS	Unadj	n/a	100	4	I: 35	ADC: 42	Current: 37%	Continuous
	CD8							II: 41	SCC: 58	Former: 52%	
								III: 24		Never: 9%	
Su, 2018 [66]	CD8	Stroma	OS & DFS	Adj	age, sex, smoking, micropapillary pattern, CEA, PD-1 expression	223	54	I: 67	ADC: 100	Smoker: 30%	≥25%
								II: 14		Non-Smoker: 70%	
								III: 19			
Matsubara, 2019 [67]	CD3	Tumor Margins	OS	Unadj and Adj	age, stage, lymphatic invasion, PD-L1, PD-L2	211	83	I: 54	SCC: 100	Smokers ≥30 pack-years: 85%	Median
	CD4							II: 35			
	CD8							III: 11			
Meng, 2019 [68]	CD4^^^	Tumor (FoxP3 Stroma)	OS	Adj	T, N, CD4, CD8, FoxP3, PD-L1	197	65	I: 57	SCC: 43	Smoker: 58%	CD4: 5%
	CD8^*^							II: 45	Non-SCC: 57	Non-smoker: 42%	CD8: 5%
	FoxP3^*^							III: 40			FoxP3: 20%
Cao, 2019 [69]	CD68	Tumor	OS & DFS	Unadj and Adj	T, N	137	56	I: 34	ADC: 68	Former or Current: 48%	Median
								II: 34	SCC: 32	Never: 53%	
								III: 33			

^*^HR reported for stage I only. ^^^HR reported for squamous only.

^#^ADC = adenocarcinoma, SCC = squamous cell carcinoma, LCC = large cell carcinoma, NR = not reported, n/a = not applicable, OS = overall survival, DFS = disease free survival, Adj = adjusted for covariates, Unadj = not adjusted for covariates.

### CD3

The CD3 complex is a defining feature of T cell lineage. T lymphocytes are an important aspect of adaptive immunity, subsets of which accomplish various immune system functions [14]. Ten eligible studies (*n* = 1,385 patients) reported on CD3+T lymphocytes as a prognostic biomarker. Of these, 7 articles reported unadjusted OS HRs (*n* = 1,108), 5 articles unadjusted DFS HR information (*n* = 415), and 3 articles reported adjusted OS HRs (*n* = 428); no articles reported an adjusted DFS HR estimate.

### CD4

CD4+ T helper cells function as part of the adaptive immune system, where they are involved in priming the immune response through their interaction with major histocompatibility complex (MHC) class II proteins on antigen presenting cells (APCs). Upon activation, they differentiate and release a variety of cytokines to help B cells make antibodies, induce macrophage activity, and recruit neutrophils, eosinophils and basophils [15]. There were 10 studies (*n* = 1,496 patients) on CD4+ T helper cells as a prognostic maker in NSCLC patients. Five articles reported unadjusted OS HR (*n* = 1,037) and 3 articles reported unadjusted DFS (*n* = 466). Six articles reported an adjusted OS HR (*n* = 699), while only 1 article gave an adjusted DFS estimate (*n* = 105).

### CD8

CD8+ cytotoxic T cells recognize antigen peptides presented on MHC class I molecules and are critical for immune defense against intracellular pathogens (i.e., viruses, bacteria) and for tumor surveillance [16]. There were 26 studies (*n* = 5,624 patients) that provided HR estimates for the association between CD8+ cytotoxic T cells and survival in stage I-III NSCLC patients. Fifteen of these articles gave unadjusted OS estimates (*n* = 3,317) and 8 reported on unadjusted DFS (*n* = 1,603). Thirteen articles gave an adjusted OS HR estimate (*n* = 2,456), while 9 reported an adjusted DFS HR (*n* = 2,596).

### CD20

CD20 is expressed on the surface of B lymphocytes, which provide humoral immunity through the production of antibodies. The specific function of tumor infiltrating B cells is still being explored, though it’s suggested that these cells play a role in anti-tumor immunity through either directly presenting antigens to T cells or by generating tumor antigen-specific antibodies that form immune complexes with tumor antigens that are presented by professional APCs [17]. Seven eligible studies (*n* = 1,058) provided HRs on CD20+ B cells as a prognostic biomarker in NSCLC; 5 reported unadjusted OS (*n* = 897) and 4 reported unadjusted DFS (*n* = 540). Only one article reported an estimate for adjusted OS (*n* = 113) or adjusted DFS (*n* = 218).

### FoxP3

T regulatory cells expressing FoxP3 are important for maintaining immune homeostasis by suppressing the proliferation and activation of cytotoxic T cell response [18]. There were 14 eligible (*n* = 2,464 patients) articles that reported on the predictive value of FoxP3+ T regulatory cells for survival; 9 articles included unadjusted OS (*n* = 1,547), 3 reported unadjusted DFS (*n* = 5080), 7 reported adjusted OS (*n* = 934) and 3 reported unadjusted DFS (*n* = 683).

### CD56 and 57 (NK Cells)

CD56/57 expressing NK cells, a part of the innate immune system, are capable of quickly recognizing and killing both infected and malignant cells that have lost their MHC I receptors, without priming or prior activation [19]. Six articles reported on survival measures for CD56+ & CD57+ NK cells (*n* = 622). Three of these reported unadjusted OS (*n* = 258) and 1 reported unadjusted DFS (*n* = 84). Four articles reported adjusted OS (*n* = 443) with 2 reporting adjusted DFS (*n* = 280).

### CD68

TAMS (as identified by CD68 antibodies) are thought to be driven by immunosuppressive cytokines such as IL-10 and TGF-beta and have been associated with suppressing T cell tumor response and promoting tumor growth and spread [20]. There were 12 eligible articles (*n* = 1,699); 6 reported unadjusted OS (*n* = 922), 1 study reported on unadjusted DFS (*n* = 137). Seven articles reported adjusted OS (*n* = 716), and 6 reported adjusted DFS (*n* = 861).

### MCs

When activated, MCs release inflammatory mediators, increasing vascular permeability and recruiting other immune cells [21]. Their role in cancer development, however, is largely unknown. There were 4 unique eligible articles (*n* = 771 patients) that investigated OS according to MC infiltration. Three unique articles reported an unadjusted OS (*n* = 591), with only one study reporting unadjusted DFS (*n* = 201). Four articles reported adjusted OS (*n* = 733), while 1 study reported adjusted DFS (*n* = 201).

### Immuno-infiltrates and survival

There were no significant differences between high vs. low CD3+ T cells or CD4+ T Helper cell infiltration in terms of OS or DFS (Table 2; Supplementary Figures 1 and 2). Adjusted data showed that increased levels of CD8+ T cytotoxic cells were predictive of improved OS (HR_OS (adj)_ = 0.68; 95% CI, 0.50–0.93) and DFS (HR_DFS (adj)_ = 0.60; 95% CI, 0.41–0.87; Table 2; Supplementary Figure 3A). However, CD8+ T cells were not significantly associated with improved survival (OS and DFS) in unadjusted analysis. Both unadjusted OS and DFS estimates indicated an association between CD20+ B cells and better survival (HR_OS (unadj)_ = 0.45; 95% CI, 0.22–0.93 and HR_DFS (unadj)_ = 0.57; 95% CI, 0.33–1.00, respectively). There was only one study reporting adjusted OS, which confirmed the finding (HR_OS (adj)_ = 0.16; 95% CI, 0.04–0.64; Table 2; Supplementary Figure 4A). High levels of FoxP3+ Treg lymphocytes were associated with worse OS when looking at both unadjusted (HR_OS (unadj)_ = 1.78; 95% CI, 1.20–2.64) and adjusted (HR_OS (adj)_ = 2.22; 95% CI, 1.47–3.34) data, and with worse DFS (HR_DFS (adj)_ = 2.07; 95% CI, 1.10–3.90) when adjusting for covariates (Table 2; Supplementary Figure 5A). Patients with a higher level of NK cells (CD56/CD57+) had significantly better adjusted OS than those with lower levels (HR_OS (adj)_ = 0.50; 95% CI, 0.26–0.95), although this association was not significant in the meta-estimate of unadjusted studies (Table 2; Supplementary Figure 6A). Increased presence of macrophages (CD68+) did not show a statistically significant prognostic value in terms of OS, although CD68+ macrophages were associated with worse adjusted DFS (HR_DFS (adj)_ = 1.84; 95% CI, 1.02–3.34), (Table 2; Supplementary Figure 7A). Higher levels of MCs were indicative of worse OS in the analysis of adjusted studies (HR_OS (adj)_ = 2.13; 95% CI, 1.14–3.96; Table 2; Supplementary Figure 8A).

**Table 2:** Unadjusted and adjusted pooled survival estimates

*Overall Survival (OS)*								
Biomarker	Unadjusted HR (95% CI)	Number of Articles	*n*	Heterogeneity	Adjusted HR (95% CI)	Number of Articles	*n*	Heterogeneity
CD3	0.99 (0.87, 1.12)	7	1,108	I^2^ = 0.00%	0.71 (0.37, 1.37)	3	428	I^2^ = 0.00%
				Q = 1.64 (*p* = 0.90)				Q = 1.76 (*p* = 0.42)
CD4	0.76 (0.44, 1.32)	5	1,037	I^2^ = 34.65%	1.00 (0.55, 1.81)	6	699	I^2^ = 33.79%
				Q = 3.47 (*p* = 0.19)				Q = 7.55 (*p* = 0.18)
CD8	0.99 (0.95, 1.04)	15	3,317	I^2^ = 0.00%	**0.68 (0.50, 0.93)**	13	2,456	I^2^ = 23.78%
				Q = 10.96 (*p* = 0.69)				Q = 15.74 (*p* = 0.20)
CD20	**0.45 (0.22, 0.93)**	5	861	I^2^ = 52.29%	**0.16 (0.04, 0.64)**	1	113	–
				Q = 8.38 (*p* = 0.08)				
FoxP3	**1.78 (1.20, 2.64)**	9	1,547	I^2^ = 14.93%	**2.22 (1.47, 3.34)**	7	934	I^2^ = 0.00%
				Q = 9.40 (*p* = 0.31)				Q = 4.74 (*p* = 0.58)
CD56/CD57	0.66 (0.35, 1.25)	3	258	I^2^ = 13.63%	**0.50 (0.26, 0.95)**	4	443	I^2^ = 0.00%
				Q = 2.32 (*p* = 0.31)				Q = 0.22 (*p* = 0.97)
CD68	1.36 (0.75, 2.45)	6^#^	922	I^2^ = 40.21%	1. 13 (0.77, 1.65)	7	716	I^2^ = 0.00%
				Q = 10.03 (*p* = 0.12)				Q = 4.18 (*p* = 0.65)
MCs	**1.81 (1.01, 3.15)**	3^^^	462	I^2^ = 0.00%	2.01 (0.88–3.92)	3	604	I^2^ = 43.99%
				Q = 0.09 (*p* = 0.90)				Q = 5.36 (*p* = 0.15)

*Disease Free Survival (DFS)*								
Biomarker	Unadjusted HR (95% CI)	Number of Articles	*n*	Heterogeneity	Adjusted HR (95% CI)	Number of Articles	*n*	Heterogeneity
CD3	0.98 (0.86, 1.12)	5	415	I^2^ = 0.00%	–	–	–	–
				Q = 2.63 (*p* = 0.62)				
CD4	0.72 (0.39, 1.32)	3	466	I^2^ = 0.00%	0.59 (0.11, 3.12)	1	105	–
				Q = 0.60 (*p* = 0.74)				
CD8	0.94 (0.77, 1.16)	8	1,630	I^2^ = 16.65%	**0.60 (0.41, 0.87)**	9	2,596	I^2^ = 20.43%
				Q = 8.39 (*p* = 0.30)				Q = 10.05 (*p* = 0.26)
CD20	**0.57 (0.33, 1.00)**	4	540	I^2^ = 0.00%	0.51 (0.20, 1.32)	1	218	–
				Q = 0.27 (*p* = 0.97)				
FoxP3	1.45 (0.81, 2.59)	3	508	I^2^ = 0.00%	**2.07 (1.10, 3.90)**	3	683	I^2^ = 0.00%
				Q = 0.07 (*p* = 0.96)				Q = 0.02 (*p* = 0.99)
CD56/CD57	1.35 (0.39, 4.66)	1	84	–	0.59 (0.27, 1.28)	2	280	I^2^ = 0.00%
								Q = 0.34 (*p* = 0.56)
CD68	1.81 (0.66, 4.92	1	137	–	**1.84 (1.02, 3.34)**	6^#^	861	I^2^ = 29.96%
								Q = 7.14 (*p* = 0.21)
MCs	2.30 (1.20–4.70)	1	201	–	1.50 (0.60, 3.60)	1	201	–

^#^6 unique articles but 7 estimates. For Parra, 2016 adenocarcinoma and squamous histologies were evaluated separately. ^^^3 unique articles but 4 estimates.

HR >1 refers to high level of immune cell subtype associated with worse survival.

## DISCUSSION

To our knowledge, this is the first meta-analysis demonstrating immune cell subpopulations within the TME having prognostic value in early-stage NSCLC patients undergoing surgical resection. Furthermore, this meta-analysis is also the first to present findings according to whether or not studies adjusted for clinical covariates. In the analysis of unadjusted studies, only CD20+ B cells were associated with improved OS and DFS, while FOXP3+ Tregs were associated with worse OS. However, when evaluating studies that adjusted for important clinical covariates (such as stage and age), higher levels of CD8+ cytotoxic T cells were associated with improved OS and DFS, and increased CD20+ B cells and CD 56/57+ NK cells were associated with improved OS. Lung cancers with increased FoxP3+ T regulatory cells or increased MCs had worse OS, and cancers with increased CD68+ macrophages had worse DFS. Our results are in keeping with what is known about the function of these immune cells; we now show these cells appear to have an important role in clinical outcomes of early-stage lung cancer.

Data evaluating the role of immune cells of the TME and lung cancer outcomes mostly come from small case series that are limited by sample size. Three prior meta-analyses attempting to combine the data from these studies have reported improved outcomes with increased CD8+ T cells and worse outcomes associated with FOXP3+ T regulatory cells [8–10]. However, the impact of CD20+ B cells, CD3+ T cells, CD4+ T cells and TAMs on survival was not consistent across studies. These meta-analyses presented significant heterogeneity by including data from both early- and advanced-stage NSCLC patients, possibly conferring different stages of the immunoediting process. Furthermore, patients with metastatic disease are subject to different treatments strategies (i.e., systemic chemotherapies, immunotherapies, targeted treatments) that can affect both the immune composition of the TME and survival outcomes. Additionally, tissue samples from metastatic disease are typically biopsies (i.e., core or fine needle aspirates), and it is unclear whether immunophenotyping of these samples completely characterizes the TME. Our study overcomes several of these limitations by focusing on the immune cell composition of resected early-stage lung cancers, enabling us to evaluate studies using 1) a more homogenous NSCLC population that is likely treatment naïve (except possibly the small percentage receiving neo-adjuvant chemotherapy) and 2) larger surgical samples, to study the immune composition of the TME in a more comprehensive way. Additionally, given that surgical resection of early-stage lung cancer represents the best chance for cure and long-term survival, the analysis of this very important subgroup is an important and innovative addition to the existing literature.

This meta-analysis is also the first to assess immune cell biomarkers in lung cancer while stratifying studies based on their adjustment for clinically relevant covariates. Patient factors such as sex and stage may influence both the immune composition of the TME and post-surgical survival, and therefore may distort the true relationship between the TME and survival. Our study in fact showed differences in the adjusted and unadjusted estimates of CD8+ cytotoxic T cells, NK cells, CD68+ macrophages, MCs and survival. Being that different studies were used in the unadjusted and adjusted analyses it is possible that this finding could be the result of differing methodological approaches instead of confounding. However, when we assessed direct comparisons between unadjusted and adjusted findings for a single study and biomarker, adjustment for clinical covariates impacted statistical significance in 41% of instances (Supplementary Table 1). Our ability to demonstrate significantly variable findings based on the adjustment of important clinical confounders highlights the need for future research to account for several clinical factors in order to determine the independent association of immune cell biomarkers and lung cancer survival, both overall and on a per patient basis. A consensus on standard protocol for these studies is especially needed as more immune cell subtypes in relation to cancer continues to be explored.

Our study has several limitations worth discussing. As with any meta-analysis, an important limitation is the possibility of publication bias, although the funnel plots were not suggestive of publication bias, with the exception of CD8+ T cells (Supplementary Figures 3B–3E, 4B–4C, 5B–5E, 6B–6C, 7B–7D, and 8B–8C). By including only stage I-IIIA NSCLC that underwent surgical resection we minimized heterogeneity. We were also unable to directly adjust for important clinical confounders of the relationships between immune cell biomarkers and survival. While early-stage patients undergoing surgical resection are typically treatment näive, in this meta-analysis we were not able to verify this. We did stratify the results according to the presence of adjustment for important clinical confounders, but this was still a limited approach as the included studies adjusted for a variety of different clinical covariates. For instance, only 27% of eligible articles adjusted for stage, 22% for sex, 16% for histology and just 7% for smoking. Available studies also used different cutoffs to separate high versus low biomarker infiltration, introducing additional inter-study variability. Despite these possible sources of variability, there was not statistically significant heterogeneity among studies as determined by the I^2^ statistic. While we attempted to investigate multiple relevant immune cell types, for some immune cell biomarkers, such as CD3+ T cells, CD20+ B cells, NK cells, TAMs and MCs, there was limited literature, thus comparisons between unadjusted and adjusted OS and DFS estimates was not possible.

Despite these limitations, by summarizing the results from several studies, we were able to overcome the limitation of small sample size observed in individual studies evaluating multiple immune cell biomarkers, and in doing so come to a more accurate estimate of their prognostic value. This meta-analysis is also the first to investigate if these immune cells are biomarkers for survival in surgically-resectable NSCLC patients, an important subgroup of patients that are likely growing as the result of screening guidelines. It is also the first to take clinical covariates into account.

Our results suggest that there are immune cells infiltrating the TME that can be considered biomarkers of survival in early-stage NSCLC. Specifically, we demonstrate the significant association of CD8+ cytotoxic T cells, CD20+B cells, NK cells and FoxP3+ T regulatory cells with survival in cases of early, resectable disease. The presence or absence of these immune cells within the TME of resected lung cancers may be used to stratify patients according to risk of recurrence and survival, with implications for who may be more likely to benefit from neo-adjuvant or adjuvant therapies [22]. Currently, there is ongoing debate about the effectiveness of immunotherapy treatment for early-stage lung cancers, with a concern that the delay in surgery to allow for immunotherapy neo-adjuvant treatment is more detrimental than beneficial to overall outcomes. However, if the immune infiltration profiles of those most likely to have disease recurrence after surgery can be identified, a patient’s immune infiltration can be used to determine which patients will receive the most benefit from either adjuvant or neo-adjuvant immunotherapy.

In conclusion, future randomized clinical trials should verify the prognostic value of CD8+ cytotoxic T cells, CD20+B cells, NK cells, CD68+ macrophages and FoxP3+ T regulatory cells and other immune components of the TME. Our meta-analysis was limited by what is published in the existing literature, but future studies should also attempt to determine the prognostic value of other immune cells that were beyond the scope of this work, including dendritic cells, Th1 cells, Th2 cells, Th17 cells and eosinophils, all of which may have an important impact on tumor immune escape and tumorigenesis, but at present are understudied [23, 24]. This will allow for clinicians to consider multi-immune cell panels when evaluating potential cancer outcomes and the need for additional treatment of patients with early-stage, surgically resected NSCLC.

## SUPPLEMENTARY MATERIALS



## References

[1] Bremnes RM , Busund LT , Kilvær TL , Andersen S , Richardsen E , Paulsen EE , Hald S , Khanehkenari MR , Cooper WA , Kao SC , Dønnem T . The Role of Tumor-Infiltrating Lymphocytes in Development, Progression, and Prognosis of Non-Small Cell Lung Cancer. J Thorac Oncol. 2016; 11:789–800. 10.1016/j.jtho.2016.01.015. 26845192

[2] Mittal D , Gubin MM , Schreiber RD , Smyth MJ . New insights into cancer immunoediting and its three component phases—elimination, equilibrium and escape. Curr Opin Immunol. 2014; 27:16–25. 10.1016/j.coi.2014.01.004. 24531241 PMC4388310

[3] Schreiber RD , Old LJ , Smyth MJ . Cancer Immunoediting: Integrating Immunity’s Roles in Cancer Suppression and Promotion. Science. 2011; 331:1565–1570. 10.1126/science.1203486. 21436444

[4] Galon J , Angell HK , Bedognetti D , Marincola FM . The continuum of cancer immunosurveillance: prognostic, predictive, and mechanistic signatures. Immunity. 2013; 39:11–26. 10.1016/j.immuni.2013.07.008. 23890060

[5] Uramoto H , Tanaka F . Recurrence after surgery in patients with NSCLC. Transl Lung Cancer Res. 2014; 3:242–249. 10.3978/j.issn.2218-6751.2013.12.05. 25806307 PMC4367696

[6] Galon J , Costes A , Sanchez-Cabo F , Kirilovsky A , Mlecnik B , Lagorce-Pagès C , Tosolini M , Camus M , Berger A , Wind P , Zinzindohoué F , Bruneval P , Cugnenc PH , et al. Type, density, and location of immune cells within human colorectal tumors predict clinical outcome. Science. 2006; 313:1960–1964. 10.1126/science.1129139. 17008531

[7] Remark R , Becker C , Gomez JE , Damotte D , Dieu-Nosjean MC , Sautès-Fridman C , Fridman WH , Powell CA , Altorki NK , Merad M , Gnjatic S . The non-small cell lung cancer immune contexture. A major determinant of tumor characteristics and patient outcome. Am J Respir Crit Care Med. 2015; 191:377–390. 10.1164/rccm.201409-1671PP. 25369536 PMC5447326

[8] Zeng DQ , Yu YF , Ou QY , Li XY , Zhong RZ , Xie CM , Hu QG . Prognostic and predictive value of tumor-infiltrating lymphocytes for clinical therapeutic research in patients with non-small cell lung cancer. Oncotarget. 2016; 7:13765–13781. 10.18632/oncotarget.7282. 26871598 PMC4924677

[9] Soo RA , Chen Z , Yan Teng RS , Tan HL , Iacopetta B , Tai BC , Soong R . Prognostic significance of immune cells in non-small cell lung cancer: meta-analysis. Oncotarget. 2018; 9:24801–24820. 10.18632/oncotarget.24835. 29872507 PMC5973851

[10] Geng Y , Shao Y , He W , Hu W , Xu Y , Chen J , Wu C , Jiang J . Prognostic Role of Tumor-Infiltrating Lymphocytes in Lung Cancer: a Meta-Analysis. Cell Physiol Biochem. 2015; 37:1560–1571. 10.1159/000438523. 26513143

[11] DerSimonian R , Laird N . Meta-analysis in clinical trials. Control Clin Trials. 1986; 7:177–188. 10.1016/0197-2456(86)90046-2. 3802833

[12] Higgins JP , Thompson SG , Deeks JJ , Altman DG . Measuring inconsistency in meta-analyses. BMJ. 2003; 327:557–560. 10.1136/bmj.327.7414.557. 12958120 PMC192859

[13] Viechtbauer W . metafor: Meta-Analysis Package for R. 2019 Available from https://CRAN.R-project.org/package=metafor.

[14] Chetty R , Gatter K . CD3: Structure, function, and role of immunostaining in clinical practice. J Pathol. 1994; 173:303–307. 10.1002/path.1711730404. 7525907

[15] Zhu J , Paul WE . CD4 T cells: fates, functions, and faults. Blood. 2008; 112:1557–1569. 10.1182/blood-2008-05-078154. 18725574 PMC2518872

[16] Zhang N , Bevan MJ . CD8+ T Cells: Foot Soldiers of the Immune System. Immunity. 2011; 35:161–168. 10.1016/j.immuni.2011.07.010. 21867926 PMC3303224

[17] Nelson BH . CD20+ B Cells: The Other Tumor-Infiltrating Lymphocytes. J Immunol. 2010; 185:4977–4982. 10.4049/jimmunol.1001323. 20962266

[18] Li Z , Li D , Tsun A , Li B . FOXP3+ regulatory T cells and their functional regulation. Cell Mol Immunol. 2015; 12:558–565. 10.1038/cmi.2015.10. 25683611 PMC4579651

[19] Vivier E , Tomasello E , Baratin M , Walzer T , Ugolini S . Functions of natural killer cells. Nat Immunol. 2008; 9:503–510. 10.1038/ni1582. 18425107

[20] Yang L , Zhang Y . Tumor-associated macrophages: from basic research to clinical application. J Hematol Oncol. 2017; 10:58. 10.1186/s13045-017-0430-2. 28241846 PMC5329931

[21] Krystel-Whittemore M , Dileepan KN , Wood JG . Mast Cell: A Multi-Functional Master Cell. Front Immunol. 2016; 6. 10.3389/fimmu.2015.00620. 26779180 PMC4701915

[22] Shahid M , Choi TG , Nguyen MN , Matondo A , Jo YH , Yoo JY , Nguyen NN , Yun HR , Kim J , Akter S , Kang I , Ha J , Maeng CH , et al. An 8-gene signature for prediction of prognosis and chemoresponse in non-small cell lung cancer. Oncotarget. 2016; 7:86561–86572. 10.18632/oncotarget.13357. 27863408 PMC5349935

[23] Faruki H , Mayhew GM , Serody JS , Hayes DN , Perou CM , Lai-Goldman M . Lung Adenocarcinoma and Squamous Cell Carcinoma Gene Expression Subtypes Demonstrate Significant Differences in Tumor Immune Landscape. J Thorac Oncol. 2017; 12:943–953. 10.1016/j.jtho.2017.03.010. 28341226 PMC6557266

[24] Gnjatic S , Bronte V , Brunet LR , Butler MO , Disis ML , Galon J , Hakansson LG , Hanks BA , Karanikas V , Khleif SN , Kirkwood JM , Miller LD , Schendel DJ , et al. Identifying baseline immune-related biomarkers to predict clinical outcome of immunotherapy. J Immunother Cancer. 2017; 5:44. 10.1186/s40425-017-0243-4. 28515944 PMC5432988

[25] Takanami I , Takeuchi K , Naruke M . Mast cell density is associated with angiogenesis and poor prognosis in pulmonary adenocarcinoma. Cancer. 2000; 88:2686–2692. 10.1002/1097-0142(20000615)88:12<2686::AID-CNCR6>3.0.CO;2-6. 10870050

[26] Pelletier MP , Edwardes MD , Michel RP , Halwani F , Morin JE . Prognostic markers in resectable non-small cell lung cancer: a multivariate analysis. Can J Surg. 2001; 44:180–188. 11407827 PMC3699112

[27] Kojima H , Shijubo N , Abe S . Thymidine phosphorylase and vascular endothelial growth factor in patients with Stage I lung adenocarcinoma. Cancer. 2002; 94:1083–1093. 10.1002/cncr.10352. 11920479

[28] Villegas FR , Coca S , Villarrubia VG , Jiménez R , Chillón MJ , Jareño J , Zuil M , Callol L . Prognostic significance of tumor infiltrating natural killer cells subset CD57 in patients with squamous cell lung cancer. Lung Cancer. 2002; 35:23–28. 10.1016/S0169-5002(01)00292-6. 11750709

[29] Pelosi G , Barisella M , Pasini F , Leon ME , Veronesi G , Spaggiari L , Fraggetta F , Iannucci A , Masullo M , Sonzogni A , Maffini F , Viale G . CD117 immunoreactivity in stage I adenocarcinoma and squamous cell carcinoma of the lung: relevance to prognosis in a subset of adenocarcinoma patients. Mod Pathol. 2004; 17:711–721. 10.1038/modpathol.3800110. 15073598

[30] Kojima H , Shijubo N , Yamada G , Ichimiya S , Abe S , Satoh M , Sato N . Clinical significance of vascular endothelial growth factor-C and vascular endothelial growth factor receptor 3 in patients with T1 lung adenocarcinoma. Cancer. 2005; 104:1668–1677. 10.1002/cncr.21366. 16116610

[31] Petersen RP , Campa MJ , Sperlazza J , Conlon D , Joshi MB , Harpole DH , Patz EF . Tumor infiltrating Foxp3+ regulatory T-cells are associated with recurrence in pathologic stage I NSCLC patients. Cancer. 2006; 107:2866–2872. 10.1002/cncr.22282. 17099880

[32] Kikuchi E , Yamazaki K , Torigoe T , Cho Y , Miyamoto M , Oizumi S , Hommura F , Dosaka-Akita H , Nishimura M . HLA class I antigen expression is associated with a favorable prognosis in early stage non-small cell lung cancer. Cancer Sci. 2007; 98:1424–1430. 10.1111/j.1349-7006.2007.00558.x. 17645781 PMC11159758

[33] Dieu-Nosjean MC , Antoine M , Danel C , Heudes D , Wislez M , Poulot V , Rabbe N , Laurans L , Tartour E , de Chaisemartin L , Lebecque S , Fridman WH , Cadranel J . Long-term survival for patients with non-small-cell lung cancer with intratumoral lymphoid structures. J Clin Oncol. 2008; 26:4410–4417. 10.1200/JCO.2007.15.0284. 18802153

[34] Shimizu K , Nakata M , Hirami Y , Yukawa T , Maeda A , Tanemoto K . Tumor-infiltrating Foxp3+ regulatory T cells are correlated with cyclooxygenase-2 expression and are associated with recurrence in resected non-small cell lung cancer. J Thorac Oncol. 2010; 5:585–590. 10.1097/JTO.0b013e3181d60fd7. 20234320

[35] da Costa Souza P , Parra ER , Atanazio MJ , da Silva OB , Noleto GS , Ab’Saber AM , de Morais Fernezlian S , Takagaki T , Capelozzi VL . Different morphology, stage and treatment affect immune cell infiltration and long-term outcome in patients with non-small-cell lung carcinoma. Histopathology. 2012; 61:587–596. 10.1111/j.1365-2559.2012.04318.x. 22716510

[36] Hanagiri T , Shigematsu Y , Shinohara S , Takenaka M , Oka S , Chikaishi Y , Nagata Y , Iwata T , Uramoto H , So T , Tanaka F . Clinical significance of the frequency of regulatory T cells in regional lymph node lymphocytes as a prognostic factor for non-small-cell lung cancer. Lung Cancer. 2013; 81:475–479. 10.1016/j.lungcan.2013.07.001. 23891508

[37] Suzuki K , Kadota K , Sima CS , Nitadori J , Rusch VW , Travis WD , Sadelain M , Adusumilli PS . Clinical impact of immune microenvironment in stage I lung adenocarcinoma: tumor interleukin-12 receptor b2 (IL-12Rb2), IL-7R, and stromal FoxP3/CD3 ratio are independent predictors of recurrence. J Clin Oncol. 2013; 31:490–498. 10.1200/JCO.2012.45.2052. 23269987 PMC3731922

[38] Feng PH , Yu CT , Wu CY , Lee MJ , Lee WH , Wang LS , Lin SM , Fu JF , Lee KY , Yen TH . Tumor-associated macrophages in stage IIIA pN2 non-small cell lung cancer after neoadjuvant chemotherapy and surgery. Am J Transl Res. 2014; 6:593–603. 25360223 PMC4212933

[39] Germain C , Gnjatic S , Tamzalit F , Knockaert S , Remark R , Goc J , Lepelley A , Becht E , Katsahian S , Bizouard G , Validire P , Damotte D , Alifano M , et al. Presence of B cells in tertiary lymphoid structures is associated with a protective immunity in patients with lung cancer. Am J Respir Crit Care Med. 2014; 189:832–844. 10.1164/rccm.201309-1611OC. 24484236

[40] Hanagiri T , Fukumoto M , Koyanagi Y , Furutani Y , Tanaka F . Regulatory T-cells and micrometastasis in lymph nodes of stage I NSCLC. Anticancer Res. 2014; 34:7185–7190. 25503147

[41] Lee MC , Buitrago DH , Kadota K , Ujiie H , Woo K , Sima CS , Travis WD , Jones DR , Adusumilli PS . The tumor immune microenvironment in octogenarians with stage I non-small cell lung cancer. Oncoimmunology. 2014; 3:e967142. 10.4161/21624011.2014.967142. 25941595 PMC4368147

[42] Djenidi F , Adam J , Goubar A , Durgeau A , Meurice G , de Montpréville V , Validire P , Besse B , Mami-Chouaib F . CD8+CD103+ tumor-infiltrating lymphocytes are tumor-specific tissue-resident memory T cells and a prognostic factor for survival in lung cancer patients. J Immunol. 2015; 194:3475–3486. 10.4049/jimmunol.1402711. 25725111

[43] Hernández-Prieto S , Romera A , Ferrer M , Subiza JL , López-Asenjo JA , Jarabo JR , Gómez AM , Molina EM , Puente J , González-Larriba JL , Hernando F , Pérez-Villamil B , Díaz-Rubio E , et al. A 50-gene signature is a novel scoring system for tumor-infiltrating immune cells with strong correlation with clinical outcome of stage I/II non-small cell lung cancer. Clin Transl Oncol. 2015; 17:330–338. 10.1007/s12094-014-1235-1. 25301404

[44] Kadota K , Nitadori JI , Ujiie H , Buitrago DH , Woo KM , Sima CS , Travis WD , Jones DR , Adusumilli PS . Prognostic Impact of Immune Microenvironment in Lung Squamous Cell Carcinoma: Tumor-Infiltrating CD10+ Neutrophil/CD20+ Lymphocyte Ratio as an Independent Prognostic Factor. J Thorac Oncol. 2015; 10:1301–1310. 10.1097/JTO.0000000000000617. 26291010 PMC4545576

[45] Kim MY , Koh J , Kim S , Go H , Jeon YK , Chung DH . Clinicopathological analysis of PD-L1 and PD-L2 expression in pulmonary squamous cell carcinoma: Comparison with tumor-infiltrating T cells and the status of oncogenic drivers. Lung Cancer. 2015; 88:24–33. 10.1016/j.lungcan.2015.01.016. 25662388

[46] Donnem T , Hald SM , Paulsen EE , Richardsen E , Al-Saad S , Kilvaer TK , Brustugun OT , Helland A , Lund-Iversen M , Poehl M , Olsen KE , Ditzel HJ , Hansen O , et al. Stromal CD8+ T-cell Density—A Promising Supplement to TNM Staging in Non-Small Cell Lung Cancer. Clin Cancer Res. 2015; 21:2635–2643. 10.1158/1078-0432.CCR-14-1905. 25680376

[47] Li Y , Sun BS , Pei B , Li CG , Zhang ZF , Yin YS , Wang CL . Osteopontin-expressing macrophages in non-small cell lung cancer predict survival. Ann Thorac Surg. 2015; 99:1140–1148. 10.1016/j.athoracsur.2014.11.054. 25725928

[48] O’Callaghan DS , Rexhepaj E , Gately K , Coate L , Delaney D , O’Donnell DM , Kay E , O’Connell F , Gallagher WM , O’Byrne KJ . Tumour islet Foxp3+ T-cell infiltration predicts poor outcome in nonsmall cell lung cancer. Eur Respir J. 2015; 46:1762–1772. 10.1183/13993003.00176-2014. 26541534

[49] Paulsen EE , Kilvaer T , Khanehkenari MR , Maurseth RJ , Al-Saad S , Hald SM , Al-Shibli K , Andersen S , Richardsen E , Busund LT , Bremnes R , Donnem T . CD45RO(+) Memory T Lymphocytes–a Candidate Marker for TNM-Immunoscore in Squamous Non-Small Cell Lung Cancer. Neoplasia. 2015; 17:839–848. 10.1016/j.neo.2015.11.004. 26678911 PMC4681889

[50] Tian C , Lu S , Fan Q , Zhang W , Jiao S , Zhao X , Wu Z , Sun L , Wang L . Prognostic significance of tumor-infiltrating CD8+ or CD3+ T lymphocytes and interleukin-2 expression in radically resected non-small cell lung cancer. Chin Med J (Engl). 2015; 128:105–110. 10.4103/0366-6999.147828. 25563322 PMC4837804

[51] Ameratunga M , Asadi K , Lin X , Walkiewicz M , Murone C , Knight S , Mitchell P , Boutros P , John T . PD-L1 and Tumor Infiltrating Lymphocytes as Prognostic Markers in Resected NSCLC. PLoS One. 2016; 11. 10.1371/journal.pone.0153954. 27104612 PMC4841565

[52] Kinoshita T , Muramatsu R , Fujita T , Nagumo H , Sakurai T , Noji S , Takahata E , Yaguchi T , Tsukamoto N , Kudo-Saito C , Hayashi Y , Kamiyama I , Ohtsuka T , et al. Prognostic value of tumor-infiltrating lymphocytes differs depending on histological type and smoking habit in completely resected non-small-cell lung cancer. Ann Oncol. 2016; 27:2117–2123. 10.1093/annonc/mdw319. 27502728

[53] Parra ER , Behrens C , Rodriguez-Canales J , Lin H , Mino B , Blando J , Zhang J , Gibbons DL , Heymach JV , Sepesi B , Swisher SG , Weissferdt A , Kalhor N , et al. Image Analysis-based Assessment of PD-L1 and Tumor-Associated Immune Cells Density Supports Distinct Intratumoral Microenvironment Groups in Non-small Cell Lung Carcinoma Patients. Clin Cancer Res. 2016; 22:6278–6289. 10.1158/1078-0432.CCR-15-2443. 27252415 PMC5558040

[54] Teng F , Meng X , Wang X , Yuan J , Liu S , Mu D , Zhu H , Kong L , Yu J . Expressions of CD8+TILs, PD-L1 and Foxp3+TILs in stage I NSCLC guiding adjuvant chemotherapy decisions. Oncotarget. 2016; 7:64318–64329. 10.18632/oncotarget.11793. 27602763 PMC5325445

[55] Usó M , Jantus-Lewintre E , Bremnes RM , Calabuig S , Blasco A , Pastor E , Borreda I , Molina-Pinelo S , Paz-Ares L , Guijarro R , Martorell M , Forteza J , Camps C , et al. Analysis of the immune microenvironment in resected non-small cell lung cancer: the prognostic value of different T lymphocyte markers. Oncotarget. 2016; 7:52849–52861. 10.18632/oncotarget.10811. 27463005 PMC5288153

[56] Yang CY , Lin MW , Chang YL , Wu CT , Yang PC . Programmed cell death-ligand 1 expression is associated with a favourable immune microenvironment and better overall survival in stage I pulmonary squamous cell carcinoma. Eur J Cancer. 2016; 57:91–103. 10.1016/j.ejca.2015.12.033. 26901614

[57] Talebian Yazdi M , van Riet S , van Schadewijk A , Fiocco M , van Hall T , Taube C , Hiemstra PS , van der Burg SH . The positive prognostic effect of stromal CD8+ tumor-infiltrating T cells is restrained by the expression of HLA-E in non-small cell lung carcinoma. Oncotarget. 2016; 7:3477–3488. 10.18632/oncotarget.6506. 26658106 PMC4823121

[58] Huang Z , Yu H , Zhang J , Jing H , Zhu W , Li X , Kong L , Xing L , Yu J , Meng X . Correlation of cancer stem cell markers and immune cell markers in resected non-small cell lung cancer. J Cancer. 2017; 8:3190–3197. 10.7150/jca.20172. 29158791 PMC5665035

[59] Kinoshita T , Kudo-Saito C , Muramatsu R , Fujita T , Saito M , Nagumo H , Sakurai T , Noji S , Takahata E , Yaguchi T , Tsukamoto N , Hayashi Y , Kaseda K , et al. Determination of poor prognostic immune features of tumour microenvironment in non-smoking patients with lung adenocarcinoma. Eur J Cancer. 2017; 86:15–27. 10.1016/j.ejca.2017.08.026. 28950145

[60] Koh J , Kim S , Kim MY , Go H , Jeon YK , Chung DH . Prognostic implications of intratumoral CD103+ tumor-infiltrating lymphocytes in pulmonary squamous cell carcinoma. Oncotarget. 2017; 8:13762–13769. 10.18632/oncotarget.14632. 28099920 PMC5355136

[61] Sepesi B , Cuentas EP , Canales JR , Behrens C , Correa AM , Vaporciyan A , Weissferdt A , Kalhor N , Moran C , Swisher S , Wistuba I . Programmed Death Cell Ligand 1 (PD-L1) Is Associated With Survival in Stage I Non-Small Cell Lung Cancer. Semin Thorac Cardiovasc Surg. 2017; 29:408–415. 10.1053/j.semtcvs.2017.05.008. 29195578

[62] Ye SL , Li XY , Zhao K , Feng T . High expression of CD8 predicts favorable prognosis in patients with lung adenocarcinoma: A cohort study. Medicine (Baltimore). 2017; 96:e6472. 10.1097/MD.0000000000006472. 28403077 PMC5403074

[63] Barua S , Fang P , Sharma A , Fujimoto J , Wistuba I , Rao AUK , Lin SH . Spatial interaction of tumor cells and regulatory T cells correlates with survival in non-small cell lung cancer. Lung Cancer. 2018; 117:73–79. 10.1016/j.lungcan.2018.01.022. 29409671 PMC6294443

[64] Jackute J , Zemaitis M , Pranys D , Sitkauskiene B , Miliauskas S , Vaitkiene S , Sakalauskas R . Distribution of M1 and M2 macrophages in tumor islets and stroma in relation to prognosis of non-small cell lung cancer. BMC Immunol. 2018; 19:3. 10.1186/s12865-018-0241-4. 29361917 PMC5781310

[65] Mazzaschi G , Madeddu D , Falco A , Bocchialini G , Goldoni M , Sogni F , Armani G , Lagrasta CA , Lorusso B , Mangiaracina C , Vilella R , Frati C , Alfieri R , et al. Low PD-1 Expression in Cytotoxic CD8+ Tumor-Infiltrating Lymphocytes Confers an Immune-Privileged Tissue Microenvironment in NSCLC with a Prognostic and Predictive Value. Clin Cancer Res. 2018; 24:407–419. 10.1158/1078-0432.CCR-17-2156. 29074606

[66] Su H , Xie H , Dai C , Ren Y , She Y , Xu L , Chen D , Xie D , Zhang L , Jiang G , Chen C . Characterization of TIM-3 expression and its prognostic value in patients with surgically resected lung adenocarcinoma. Lung Cancer. 2018; 121:18–24. 10.1016/j.lungcan.2018.04.009. 29858021

[67] Matsubara T , Takada K , Azuma K , Takamori S , Toyokawa G , Haro A , Osoegawa A , Tagawa T , Kawahara A , Akiba J , Okamoto I , Nakanishi Y , Oda Y , et al. A Clinicopathological and Prognostic Analysis of PD-L2 Expression in Surgically Resected Primary Lung Squamous Cell Carcinoma. Ann Surg Oncol. 2019; 26:1925–1933. 10.1245/s10434-019-07257-3. 30815803

[68] Meng X , Gao Y , Yang L , Jing H , Teng F , Huang Z , Xing L . Immune Microenvironment Differences Between Squamous and Non-squamous Non-small-cell Lung Cancer and Their Influence on the Prognosis. Clin Lung Cancer. 2019; 20:48–58. 10.1016/j.cllc.2018.09.012. 30341017

[69] Cao L , Che X , Qiu X , Li Z , Yang B , Wang S , Hou K , Fan Y , Qu X , Liu Y . M2 macrophage infiltration into tumor islets leads to poor prognosis in non-small-cell lung cancer. Cancer Manag Res. 2019; 11:6125–6138. 10.2147/CMAR.S199832. 31308749 PMC6613613

