# Supplementary material for: Prognostic value of immune cells in the tumor microenvironment of early-stage lung cancer: a meta-analysis
Source: Oncotarget. 2019 Dec 24;10(67):7142–55. doi: 10.18632/oncotarget.27392 (PMC6935257; doi:10.18632/oncotarget.27392)
Supplement: Supplementary file 1 [file oncotarget-10-7142-s001.pdf]

# Prognostic value of immune cells in the tumor microenvironment of early-stage lung cancer: a meta-analysis

## SUPPLEMENTARY MATERIALS

**Supplementary Table 1: Reported unadjusted vs. adjusted survival estimates**

| Name | Year | Biomarker | Survival Type | Unadjusted HR (95% CI) | Adjusted HR (95% CI) |
|---|---|---|---|---|---|
| O'Callaghan | 2015 | CD3 | OS | 0.70 (0.47–1.05) | 0.65 (0.41–1.02) |
| Sepesi | 2017 | CD3 | OS | 0.60 (0.32–1.12) | 0.35 (0.11–1.09) |
| **Sepesi** | **2017** | **CD4** | **OS** | **0.49 (0.29–0.85)** | **0.35 (0.11–1.09)** |
| Matsubara | 2019 | CD4 | OS | 1.55 (1.03–2.37) | 1.65 (1.07–2.55) |
| O'Callaghan | 2015 | CD8 | OS | 0.44 (0.29–0.67) | 0.48 (0.30–0.76) |
| Yazdi | 2016 | CD8 | OS | 0.64 (0.40–1.04) | 0.62 (0.38–1.01) |
| **Ameratunga** | **2016** | **CD8** | **DFS** | **0.82 (0.64–1.04)** | **0.70 (0.50–0.97)** |
| **Teng** | **2016** | **CD8** | **OS** | **0.51 (0.29–0.98)** | **0.54 (0.28–1.05)** |
| Teng | 2016 | CD8 | DFS | 0.39 (0.22–0.71) | 0.22 (0.05–0.89) |
| **Ye** | **2017** | **CD8** | **OS** | **0.71 (0.52–0.99)** | **0.70 (0.44–1.10)** |
| Kinoshita | 2017 | CD8 | DFS | 2.92 (1.69–5.07) | 2.01 (1.14–3.56) |
| Sepesi | 2017 | CD8 | OS | 1.88 (0.82–3.34) | 1.70 (0.40–7.45) |
| Kinoshita | 2016 | CD20 | DFS | 0.53 (0.34–0.84) | 0.51 (0.32–0.80) |
| Villegas | 2002 | CD57 | OS | 0.43 (0.20–0.95) | 0.40 (0.17–0.93) |
| Sepesi | 2017 | CD57 | OS | 0.24 (0.03–1.70) | 0.60 (0.05–6.51) |
| Kojima | 2002 | CD68 | OS | 1.45 (0.71–2.97) | 1.43 (0.66–3.09) |
| **Sepesi** | **2017** | **CD68** | **OS** | **0.35 (0.12–0.97)** | **0.79 (0.11–5.63)** |
| Cao | 2019 | CD68 | OS | 4.19 (1.90–9.27) | 2.86 (1.13–7.26) |
| Cao | 2019 | CD68 | DFS | 2.09 (1.26–3.46) | 1.81 (1.09–3.02) |
| **Hanagiri** | **2013** | **FoxP3** | **OS** | **2.56 (1.28–5.10)** | **1.82 (0.90–3.69)** |
| Hanagiri | 2014 | FoxP3 | OS | 2.82 (1.00–7.93) | 4.22 (1.11–5.56) |
| O'Callaghan | 2015 | FoxP3 | OS | 4.86 (3.11–7.59) | 3.91 (2.33–6.55) |
| **Barua** | **2018** | **FoxP3** | **OS** | **1.43 (0.63–3.21)** | **1.52 (1.11–2.07)** |
| **Kojima** | **2002** | **Mast Cells** | **OS** | **2.31 (1.09–4.89)** | **1.16 (0.51–2.67)** |
| **Pelosi** | **2004** | **Mast Cells** | **OS** | **2.30 (1.20–4.90)** | **1.70 (0.60–4.60)** |
| **Pelosi** | **2004** | **Mast Cells** | **DFS** | **2.30 (1.20–4.70)** | **1.50 (0.60–3.60)** |
| **Kojima** | **2005** | **Mast Cells** | **OS** | **2.04 (1.05–3.96)** | **1.15 (0.53–2.48)** |

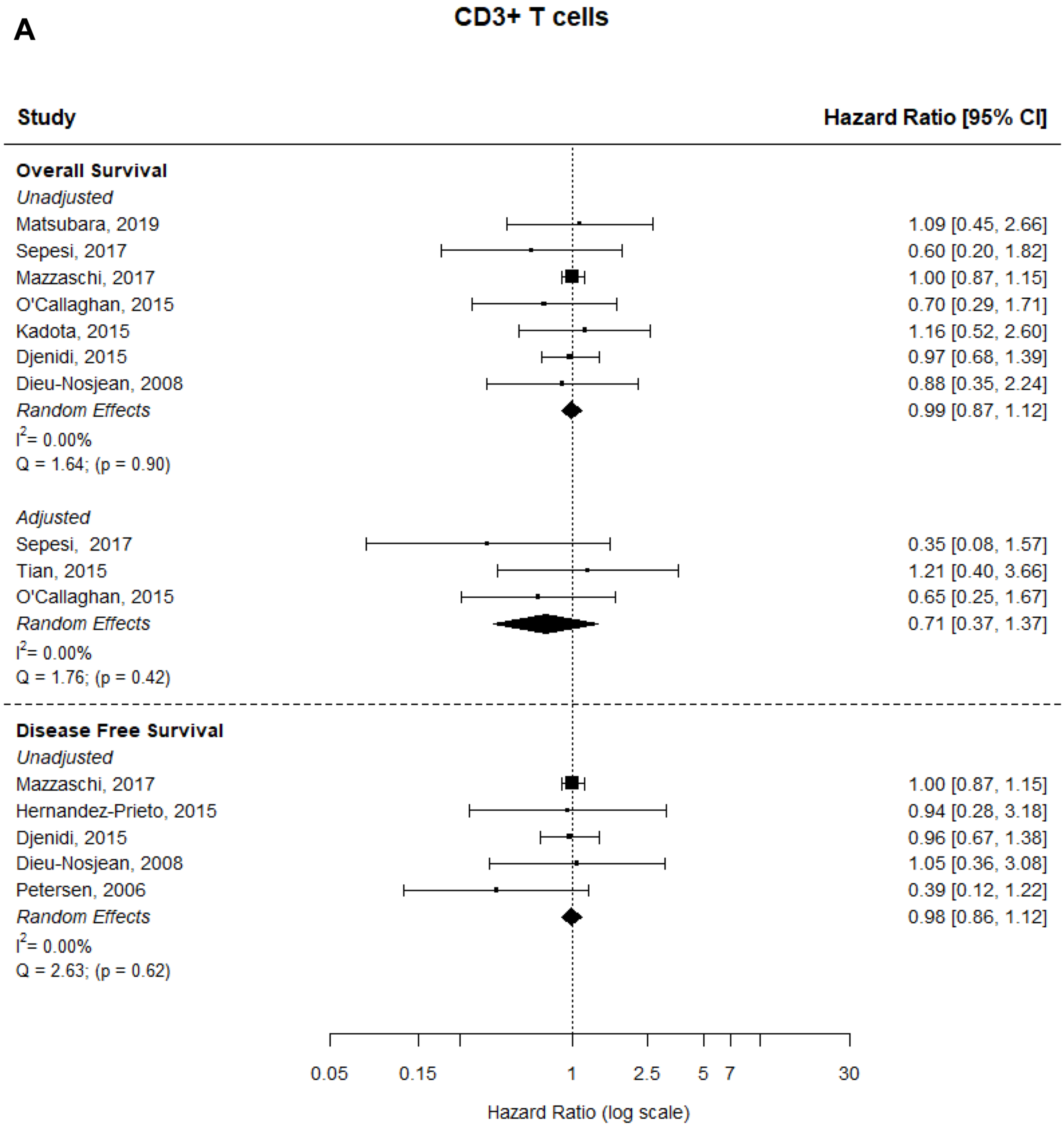
A
CD3+ T cells
Study
Hazard Ratio [95% CI]
Overall Survival
Unadjusted
Matsubara, 2019 1.09 [0.45, 2.66]
Sepesi, 2017 0.60 [0.20, 1.82]
Mazzaschi, 2017 1.00 [0.87, 1.15]
O'Callaghan, 2015 0.70 [0.29, 1.71]
Kadota, 2015 1.16 [0.52, 2.60]
Djenidi, 2015 0.97 [0.68, 1.39]
Dieu-Nosjean, 2008 0.88 [0.35, 2.24]
Random Effects 0.99 [0.87, 1.12]
I2= 0.00%
Q = 1.64; (p = 0.90)
Adjusted
Sepesi, 2017 0.35 [0.08, 1.57]
Tian, 2015 1.21 [0.40, 3.66]
O'Callaghan, 2015 0.65 [0.25, 1.67]
Random Effects 0.71 [0.37, 1.37]
I2= 0.00%
Q = 1.76; (p = 0.42)
Disease Free Survival
Unadjusted
Mazzaschi, 2017 1.00 [0.87, 1.15]
Hernandez-Prieto, 2015 0.94 [0.28, 3.18]
Djenidi, 2015 0.96 [0.67, 1.38]
Dieu-Nosjean, 2008 1.05 [0.36, 3.08]
Petersen, 2006 0.39 [0.12, 1.22]
Random Effects 0.98 [0.86, 1.12]
I2= 0.00%
Q = 2.63; (p = 0.62)
0.05
0.15
1
2.5
5
7
30
Hazard Ratio (log scale)

**B**

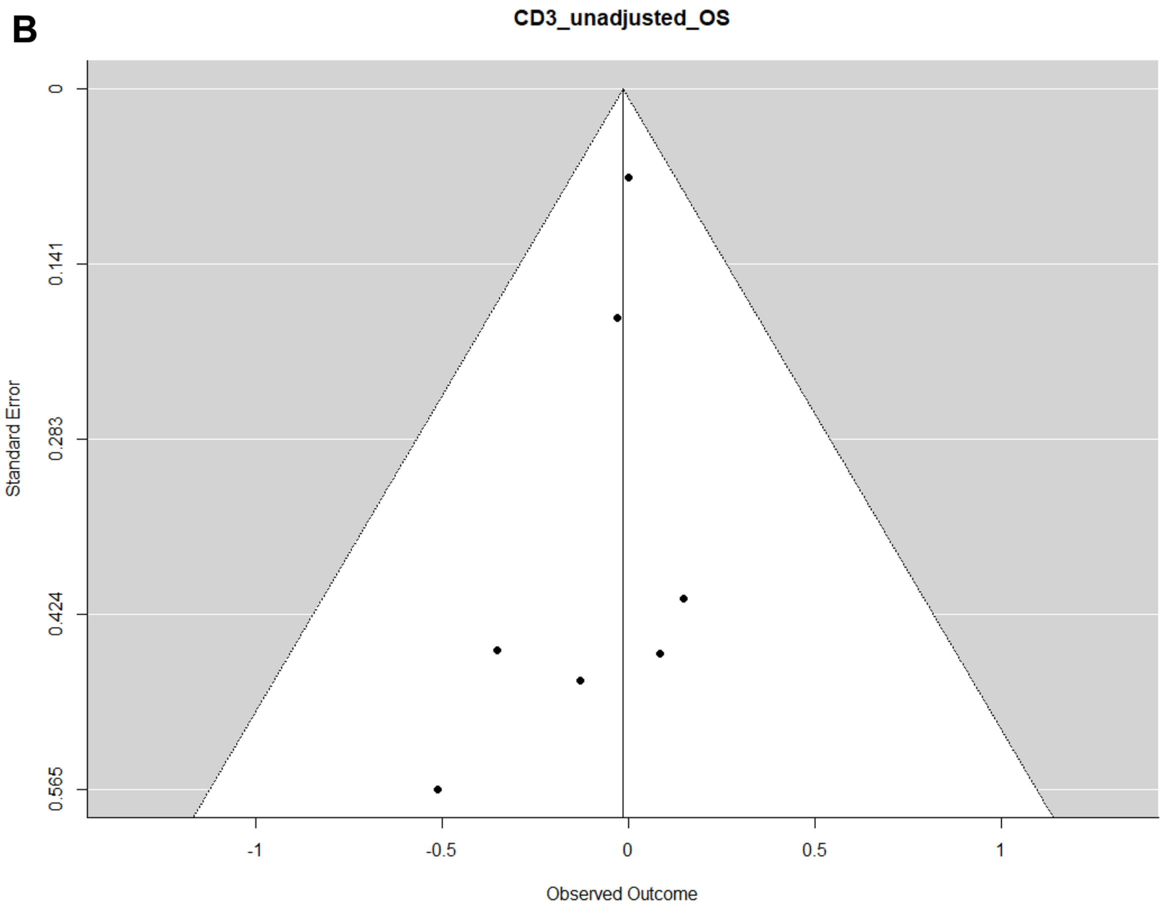
CD3_unadjusted_OS
Standard Error
0
0.141
0.283
0.424
0.565
-1
-0.5
0
0.5
1
Observed Outcome

**C**

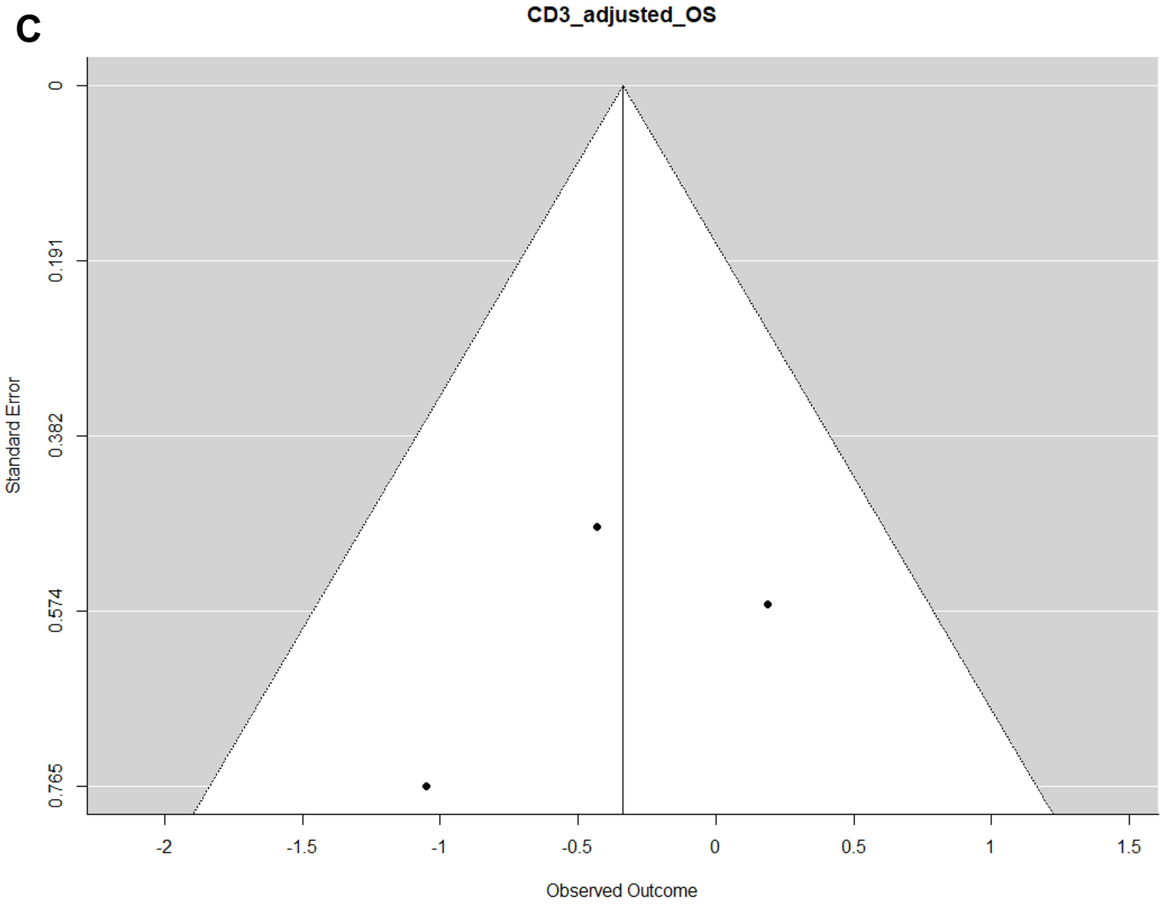
CD3_adjusted_OS
Standard Error
0
0.191
0.382
0.574
0.765
-2
-1.5
-1
-0.5
0
0.5
1
1.5
Observed Outcome

**D**

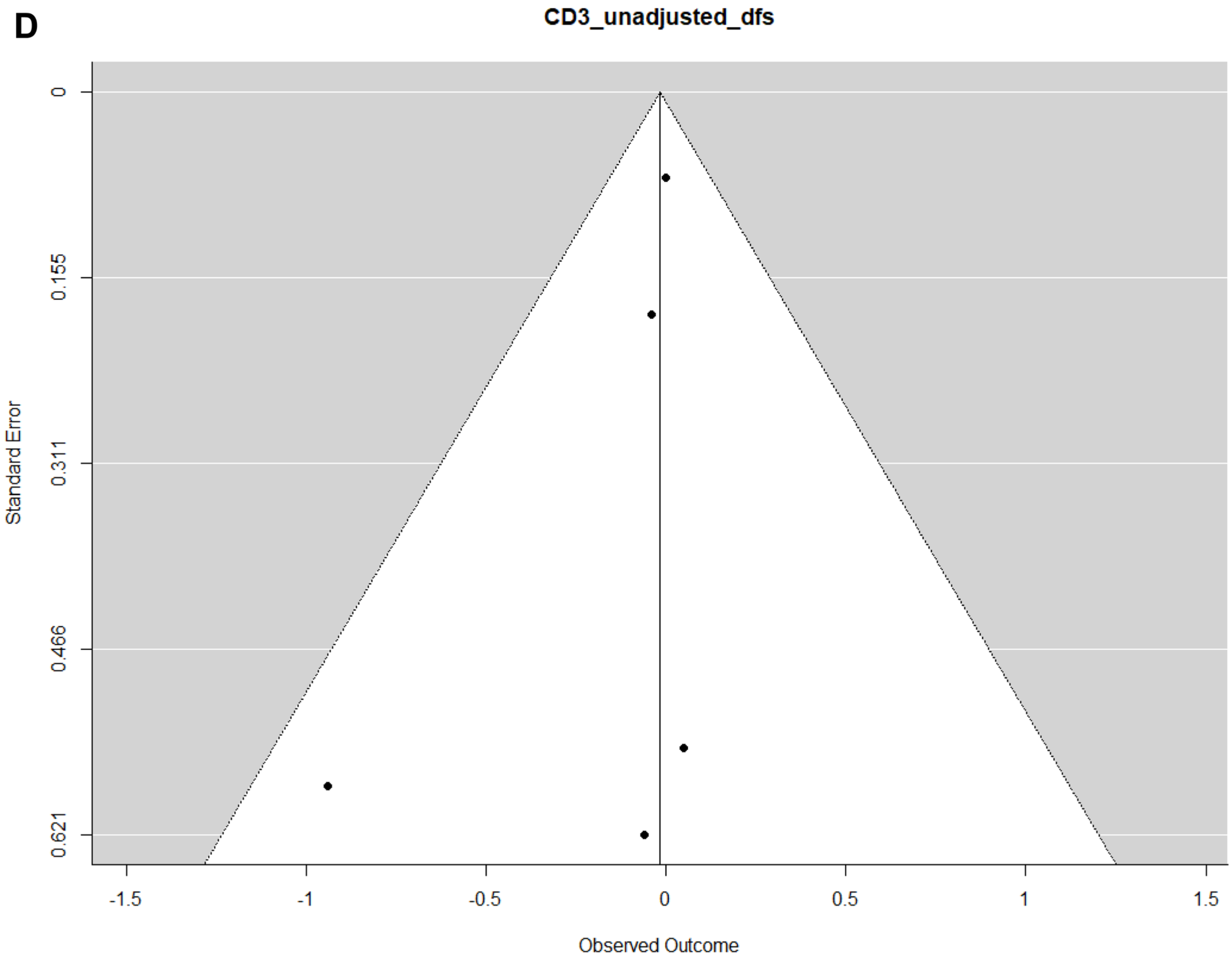


**Supplementary Figure 1:** (**A**) Forest Plot of CD3+T Cells. (**B**) Funnel Plot of CD3+T Cells Unadjusted OS Studies. (**C**) Funnel Plot of CD3+T Cells Adjusted OS Studies. (**D**) Funnel Plot of CD3+T Cells Unadjusted DFS Studies.

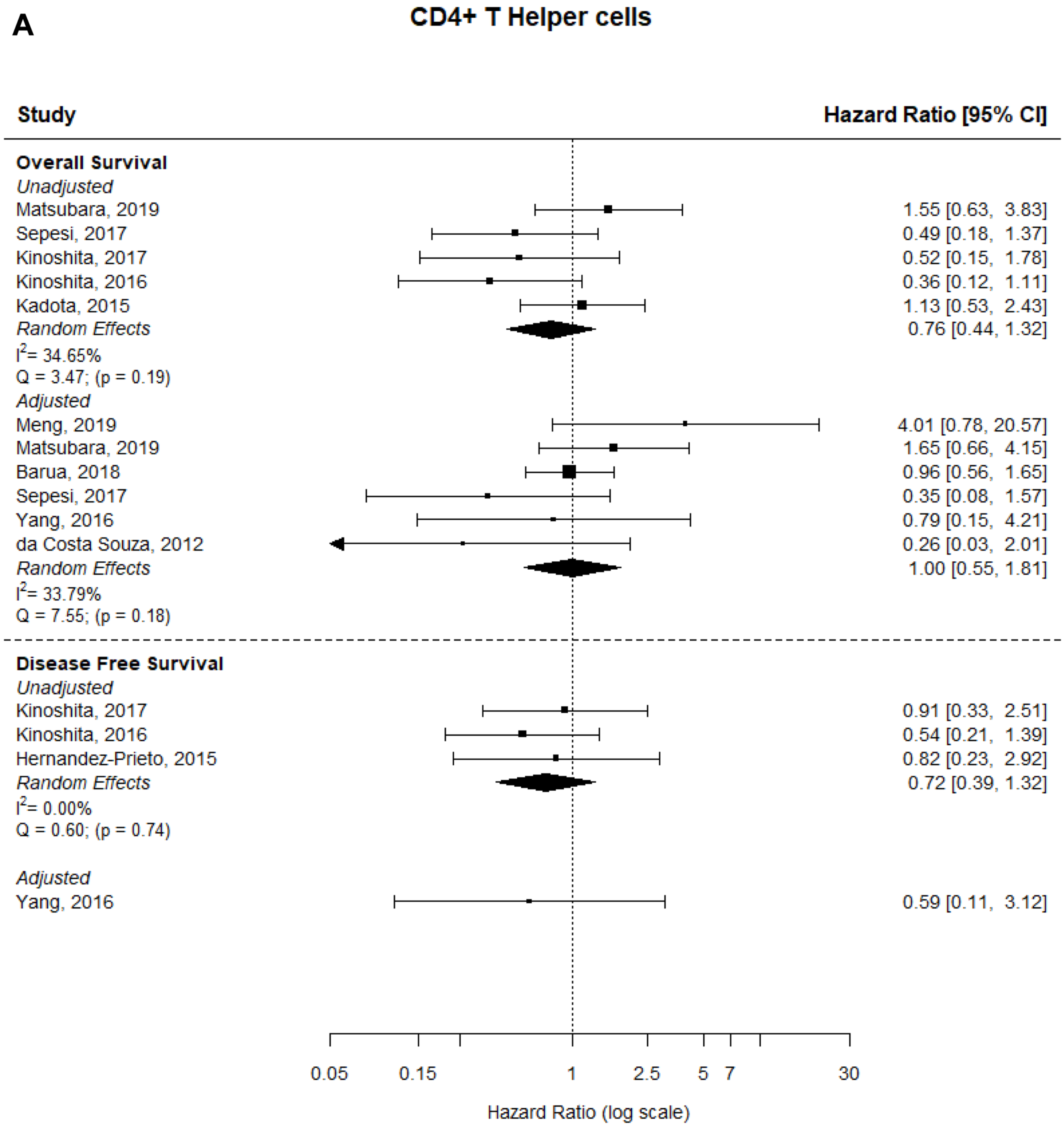
A
CD4+ T Helper cells
Study
Hazard Ratio [95% CI]
Overall Survival
Unadjusted
Matsubara, 2019
1.55 [0.63, 3.83]
Sepesi, 2017
0.49 [0.18, 1.37]
Kinoshita, 2017
0.52 [0.15, 1.78]
Kinoshita, 2016
0.36 [0.12, 1.11]
Kadota, 2015
1.13 [0.53, 2.43]
Random Effects
0.76 [0.44, 1.32]
I2= 34.65%
Q = 3.47; (p = 0.19)
Adjusted
Meng, 2019
4.01 [0.78, 20.57]
Matsubara, 2019
1.65 [0.66, 4.15]
Barua, 2018
0.96 [0.56, 1.65]
Sepesi, 2017
0.35 [0.08, 1.57]
Yang, 2016
0.79 [0.15, 4.21]
da Costa Souza, 2012
0.26 [0.03, 2.01]
Random Effects
1.00 [0.55, 1.81]
I2= 33.79%
Q = 7.55; (p = 0.18)
Disease Free Survival
Unadjusted
Kinoshita, 2017
0.91 [0.33, 2.51]
Kinoshita, 2016
0.54 [0.21, 1.39]
Hernandez-Prieto, 2015
0.82 [0.23, 2.92]
Random Effects
0.72 [0.39, 1.32]
I2= 0.00%
Q = 0.60; (p = 0.74)
Adjusted
Yang, 2016
0.59 [0.11, 3.12]
0.05
0.15
1
2.5
5
7
30
Hazard Ratio (log scale)

B

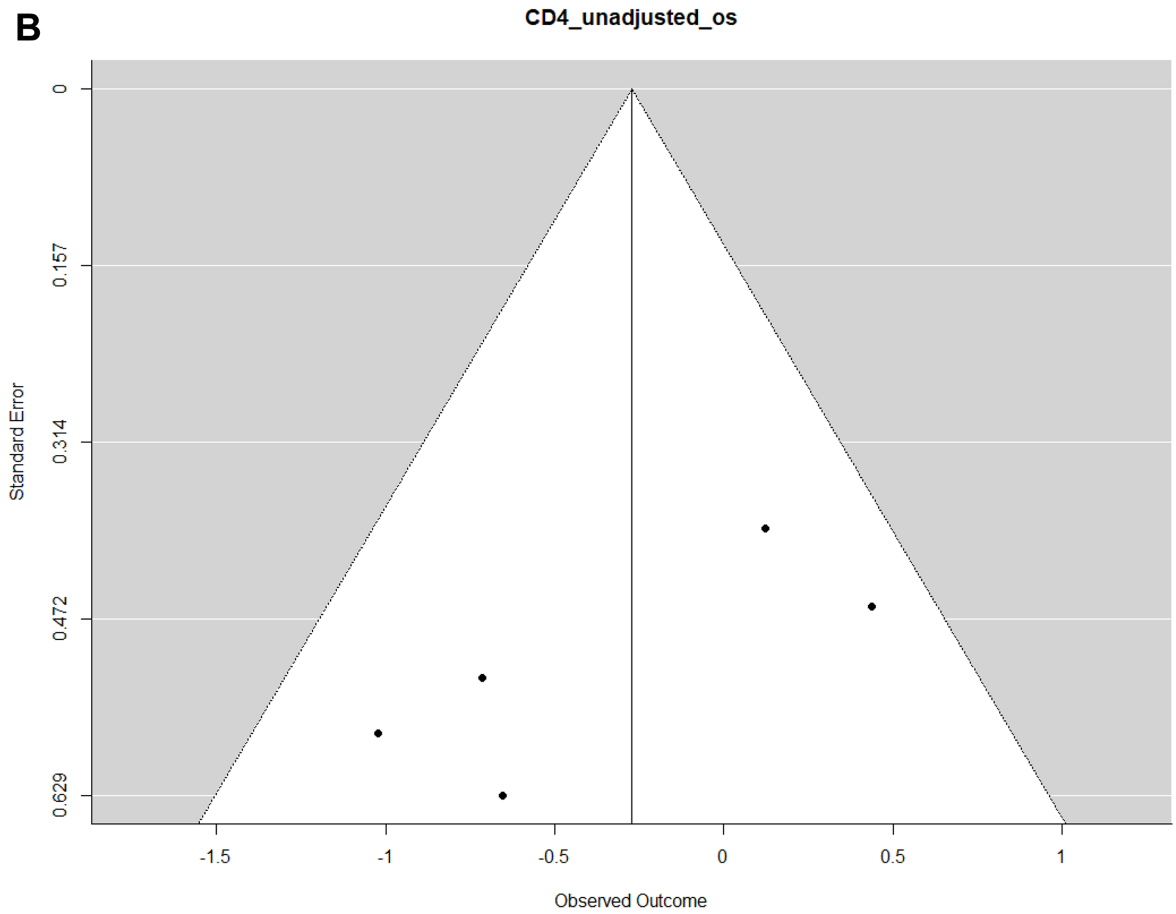
CD4_unadjusted_os
Standard Error
0
0.157
0.314
0.472
0.629
-1.5
-1
-0.5
0
0.5
1
Observed Outcome

C

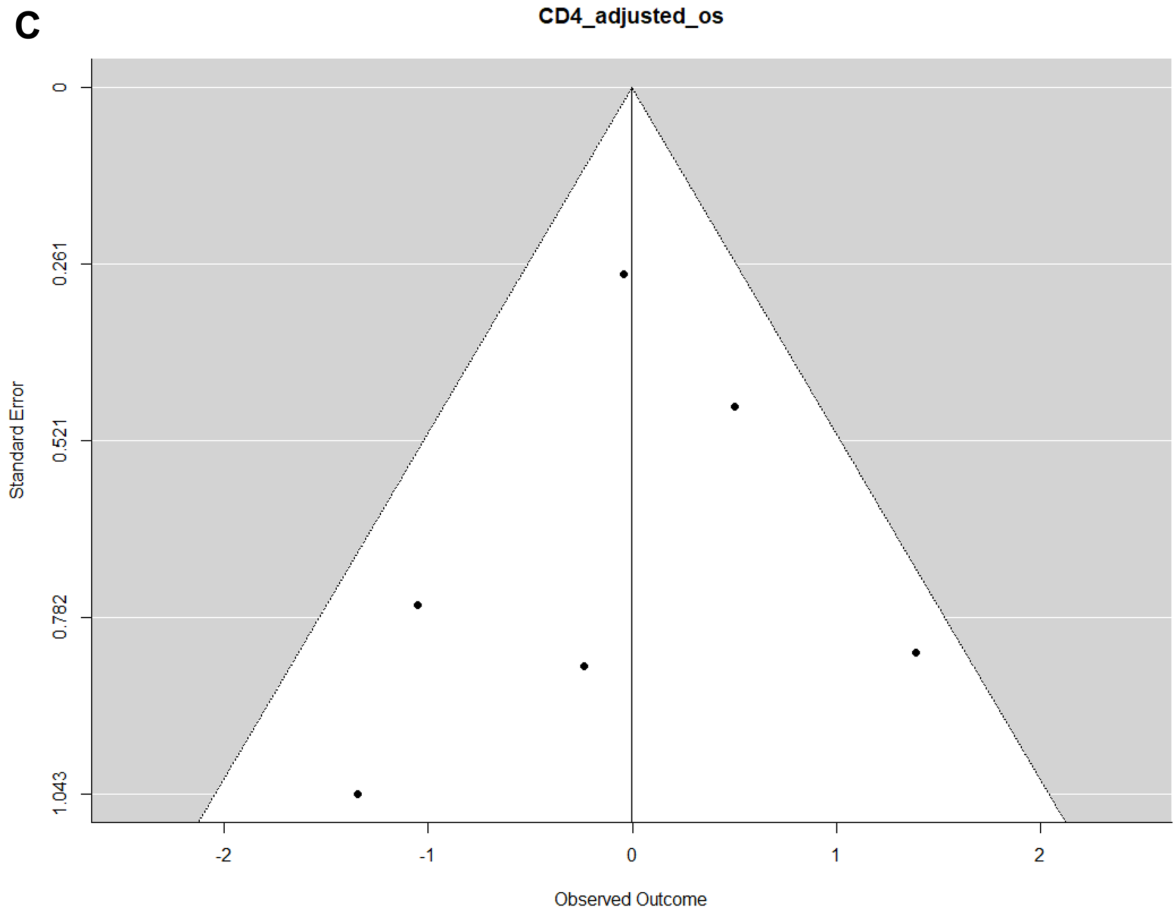
CD4_adjusted_os
Standard Error
0
0.261
0.521
0.782
1.043
-2
-1
0
1
2
Observed Outcome

D

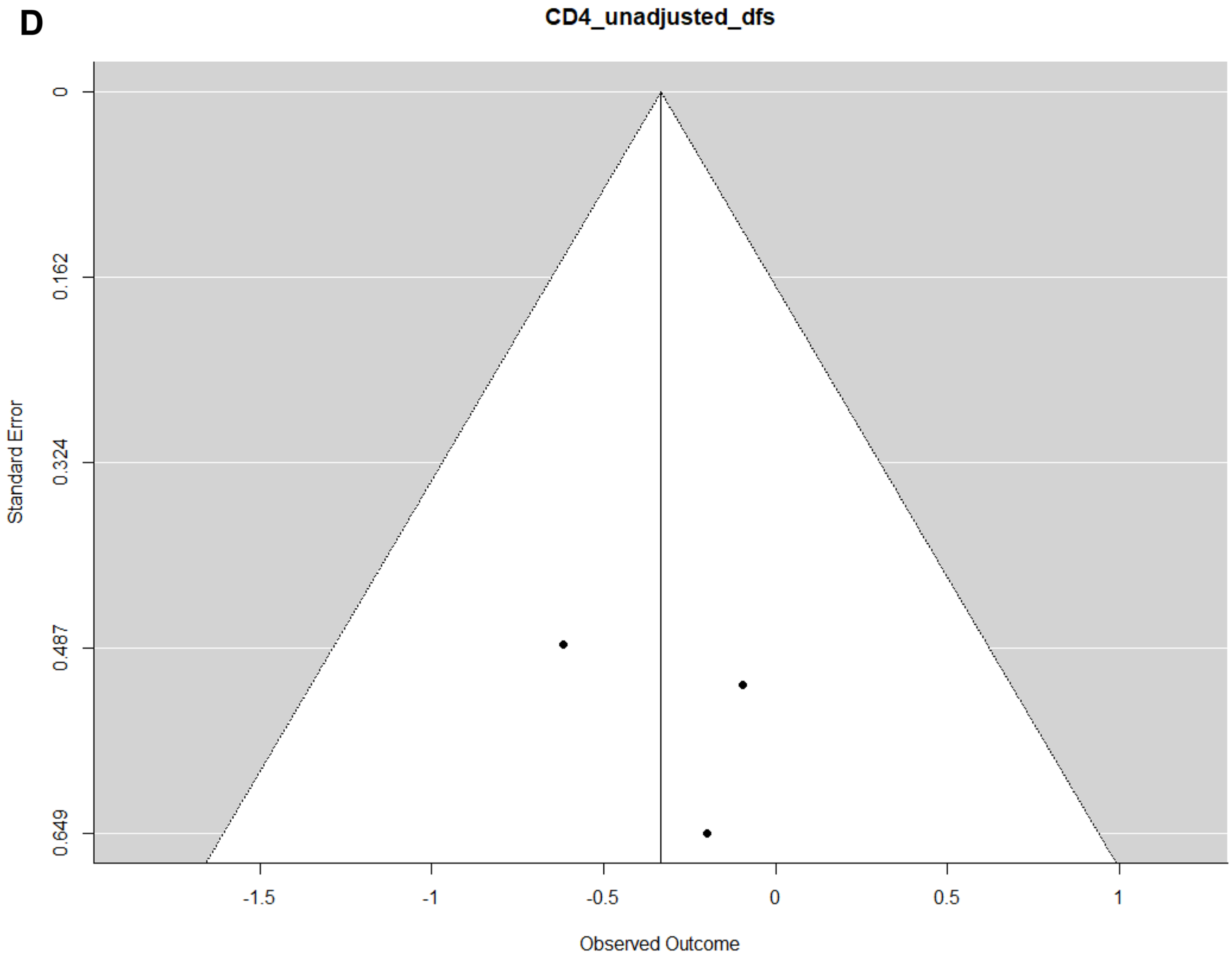


**Supplementary Figure 2:** (**A**) Forest Plot of CD4+ T Helper cells. (**B**) Funnel Plot of CD4+ T Helper cells Unadjusted OS Studies. (**C**) Funnel Plot of CD4+ T Helper cells Adjusted OS Studies. (**D**) Funnel Plot of CD4+ T Helper cells Unadjusted DFS Studies.

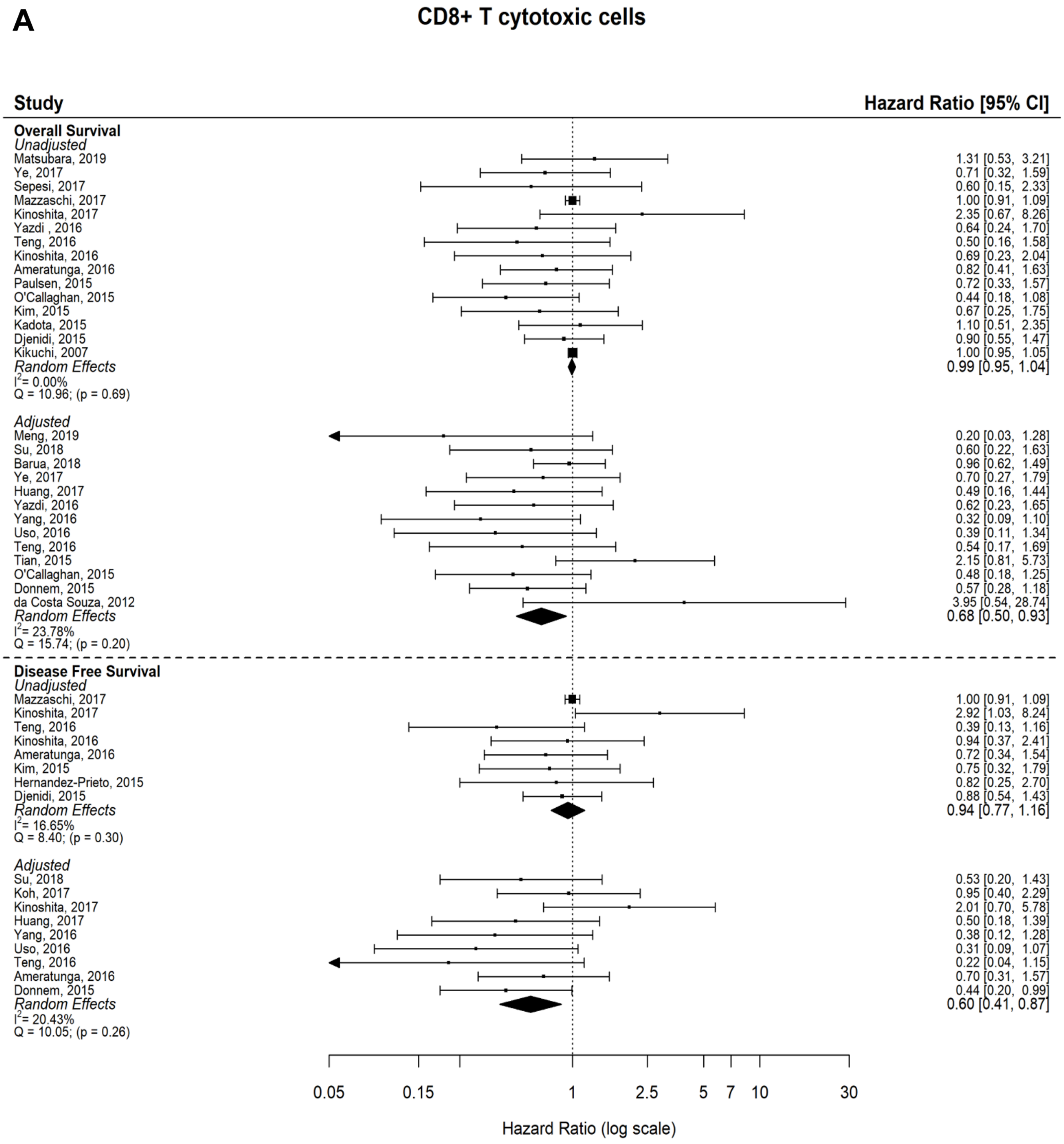
A
CD8+ T cytotoxic cells
Study
Hazard Ratio [95% CI]
Overall Survival
Unadjusted
Matsubara, 2019 1.31 [0.53, 3.21]
Ye, 2017 0.71 [0.32, 1.59]
Sepesi, 2017 0.60 [0.15, 2.33]
Mazzaschi, 2017 1.00 [0.91, 1.09]
Kinoshita, 2017 2.35 [0.67, 8.26]
Yazdi , 2016 0.64 [0.24, 1.70]
Teng, 2016 0.50 [0.16, 1.58]
Kinoshita, 2016 0.69 [0.23, 2.04]
Ameratunga, 2016 0.82 [0.41, 1.63]
Paulsen, 2015 0.72 [0.33, 1.57]
O'Callaghan, 2015 0.44 [0.18, 1.08]
Kim, 2015 0.67 [0.25, 1.75]
Kadota, 2015 1.10 [0.51, 2.35]
Djenidi, 2015 0.90 [0.55, 1.47]
Kikuchi, 2007 1.00 [0.95, 1.05]
Random Effects 0.99 [0.95, 1.04]
$I^2$= 0.00%
Q = 10.96; (p = 0.69)
Adjusted
Meng, 2019 0.20 [0.03, 1.28]
Su, 2018 0.60 [0.22, 1.63]
Barua, 2018 0.96 [0.62, 1.49]
Ye, 2017 0.70 [0.27, 1.79]
Huang, 2017 0.49 [0.16, 1.44]
Yazdi, 2016 0.62 [0.23, 1.65]
Yang, 2016 0.32 [0.09, 1.10]
Uso, 2016 0.39 [0.11, 1.34]
Teng, 2016 0.54 [0.17, 1.69]
Tian, 2015 2.15 [0.81, 5.73]
O'Callaghan, 2015 0.48 [0.18, 1.25]
Donnem, 2015 0.57 [0.28, 1.18]
da Costa Souza, 2012 3.95 [0.54, 28.74]
Random Effects 0.68 [0.50, 0.93]
$I^2$= 23.78%
Q = 15.74; (p = 0.20)
Disease Free Survival
Unadjusted
Mazzaschi, 2017 1.00 [0.91, 1.09]
Kinoshita, 2017 2.92 [1.03, 8.24]
Teng, 2016 0.39 [0.13, 1.16]
Kinoshita, 2016 0.94 [0.37, 2.41]
Ameratunga, 2016 0.72 [0.34, 1.54]
Kim, 2015 0.75 [0.32, 1.79]
Hernandez-Prieto, 2015 0.82 [0.25, 2.70]
Djenidi, 2015 0.88 [0.54, 1.43]
Random Effects 0.94 [0.77, 1.16]
$I^2$= 16.65%
Q = 8.40; (p = 0.30)
Adjusted
Su, 2018 0.53 [0.20, 1.43]
Koh, 2017 0.95 [0.40, 2.29]
Kinoshita, 2017 2.01 [0.70, 5.78]
Huang, 2017 0.50 [0.18, 1.39]
Yang, 2016 0.38 [0.12, 1.28]
Uso, 2016 0.31 [0.09, 1.07]
Teng, 2016 0.22 [0.04, 1.15]
Ameratunga, 2016 0.70 [0.31, 1.57]
Donnem, 2015 0.44 [0.20, 0.99]
Random Effects 0.60 [0.41, 0.87]
$I^2$= 20.43%
Q = 10.05; (p = 0.26)
0.05
0.15
1
2.5
5
7
10
30
Hazard Ratio (log scale)

B

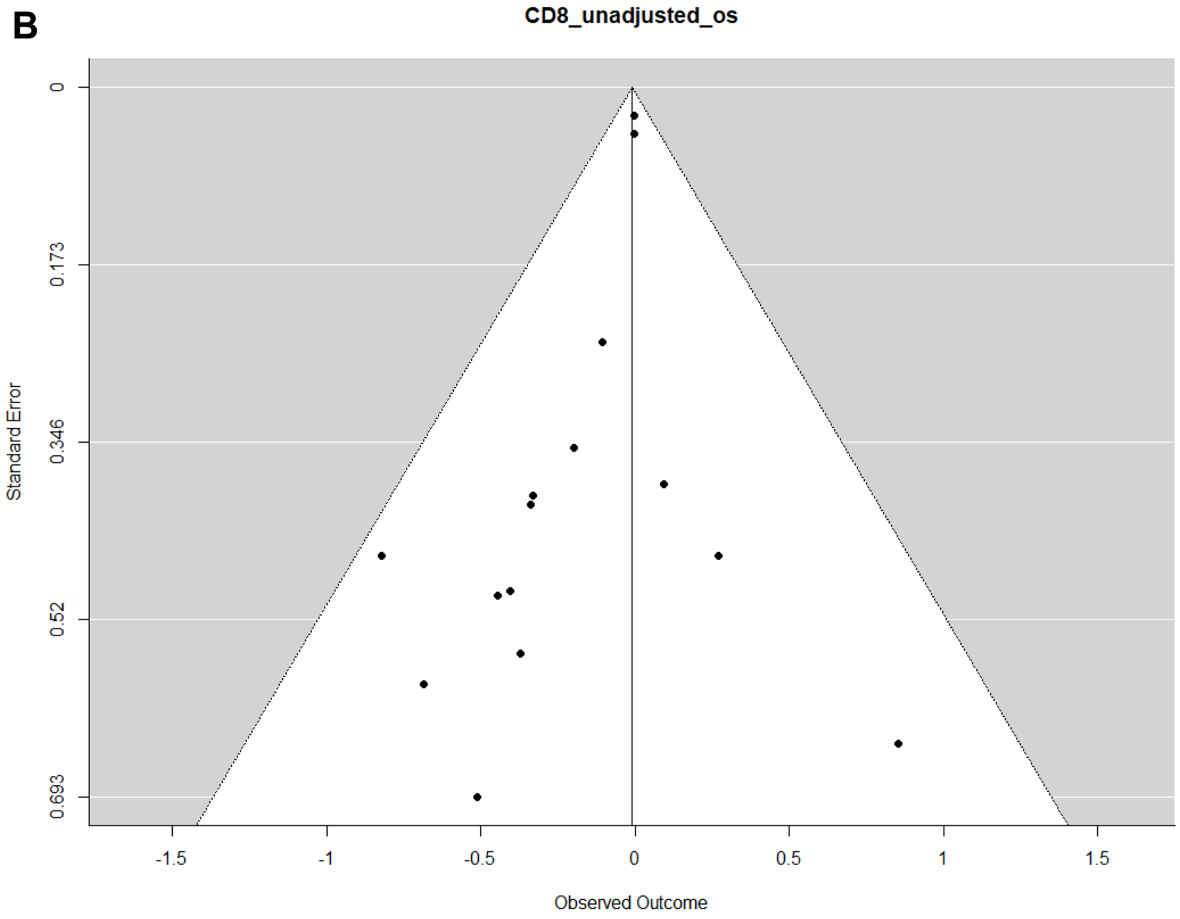
CD8_unadjusted_os
Standard Error
0
0.173
0.346
0.52
0.693
-1.5
-1
-0.5
0
0.5
1
1.5
Observed Outcome

C

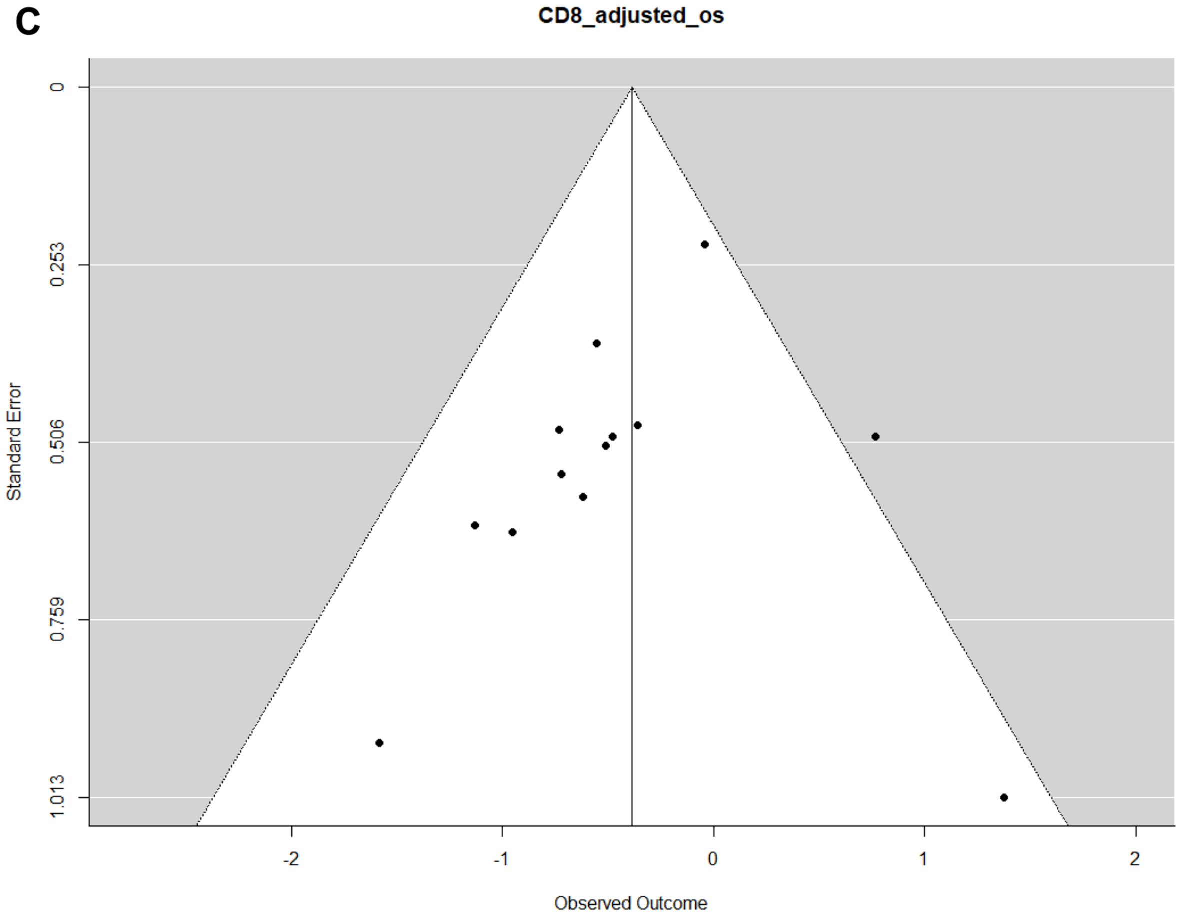
CD8_adjusted_os
Standard Error
0
0.253
0.506
0.759
1.013
-2
-1
0
1
2
Observed Outcome

D

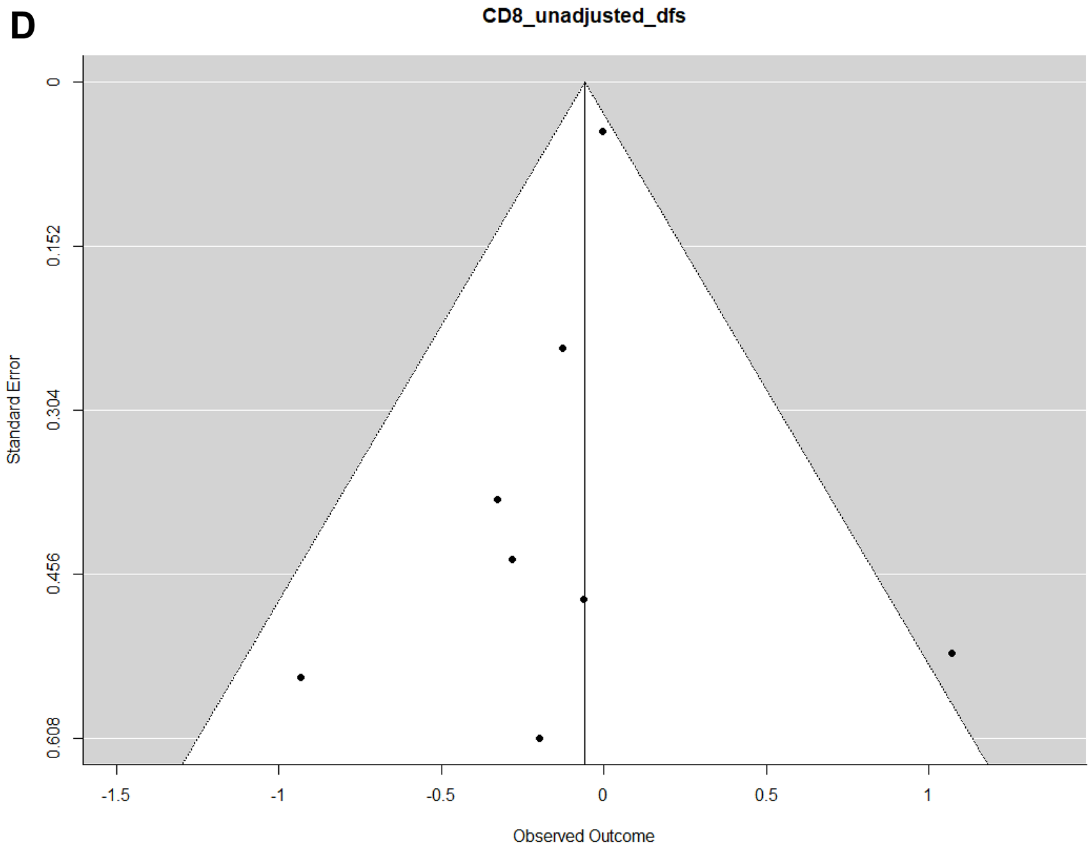


E

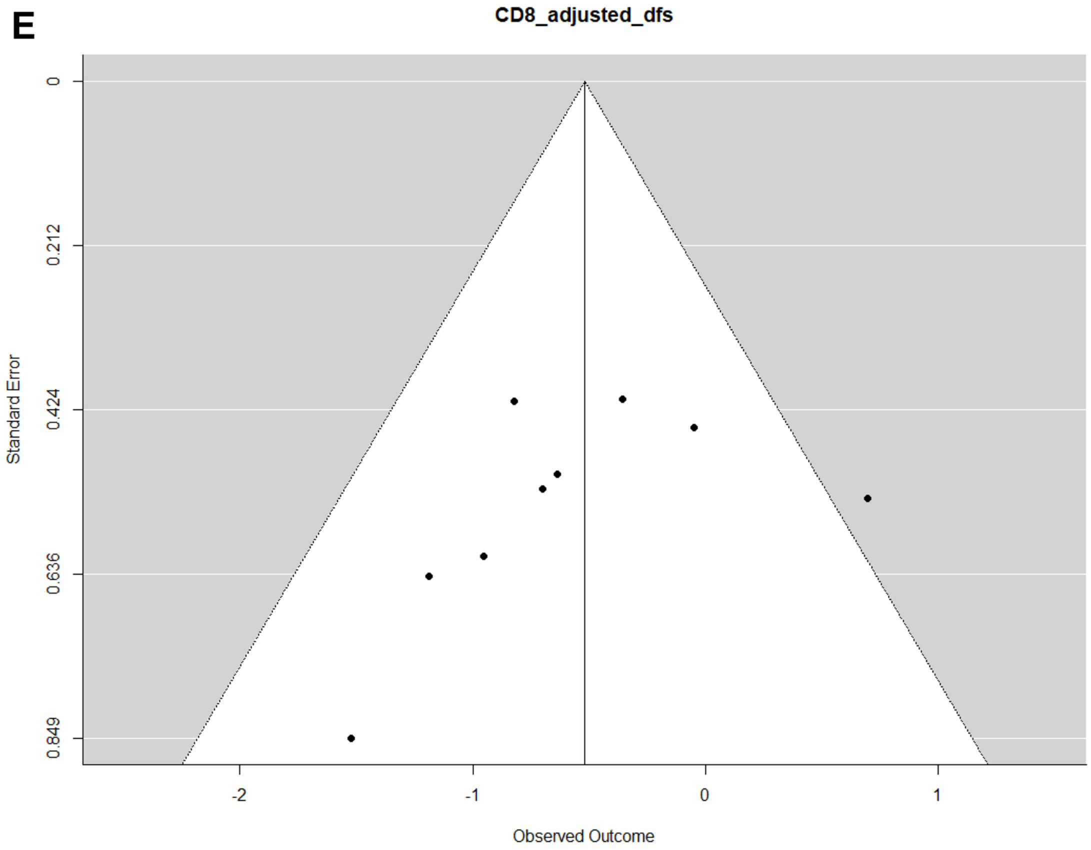


**Supplementary Figure 3:** (**A**) Forest Plot of CD8+ T cytotoxic cells. (**B**) Funnel Plot of CD8+ T cytotoxic cells Unadjusted OS Studies. (**C**) Funnel Plot of CD8+ T cytotoxic cells Adjusted OS Studies. (**D**) Funnel Plot of CD8+ T cytotoxic cells Unadjusted DFS Studies. (**E**) Funnel Plot of CD8+ T cytotoxic cells Adjusted DFS Studies.

A

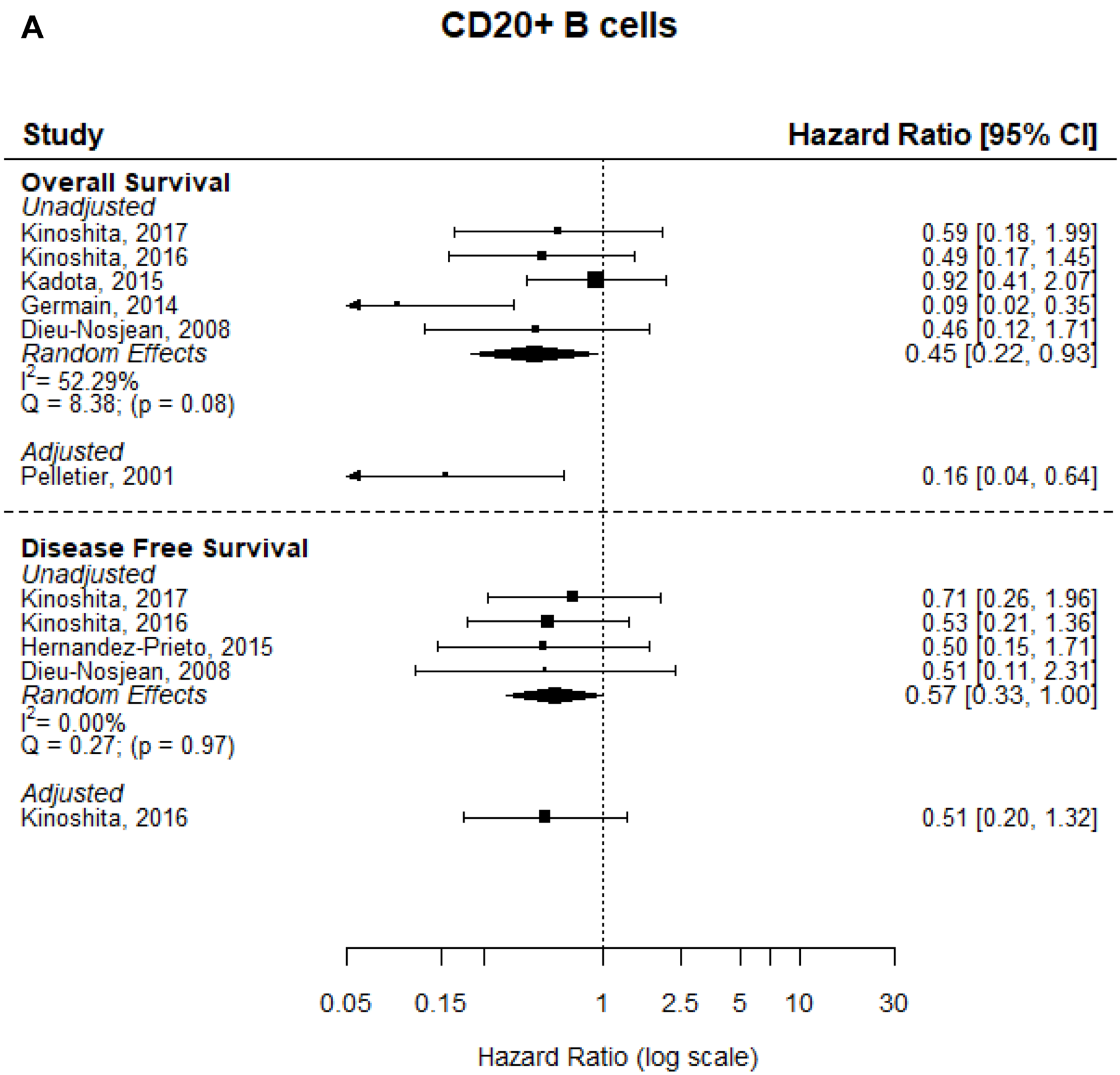
CD20+ B cells
Study
Hazard Ratio [95% CI]
Overall Survival
Unadjusted
Kinoshita, 2017 0.59 [0.18, 1.99]
Kinoshita, 2016 0.49 [0.17, 1.45]
Kadota, 2015 0.92 [0.41, 2.07]
Germain, 2014 0.09 [0.02, 0.35]
Dieu-Nosjean, 2008 0.46 [0.12, 1.71]
Random Effects 0.45 [0.22, 0.93]
I2= 52.29%
Q = 8.38; (p = 0.08)
Adjusted
Pelletier, 2001 0.16 [0.04, 0.64]
Disease Free Survival
Unadjusted
Kinoshita, 2017 0.71 [0.26, 1.96]
Kinoshita, 2016 0.53 [0.21, 1.36]
Hernandez-Prieto, 2015 0.50 [0.15, 1.71]
Dieu-Nosjean, 2008 0.51 [0.11, 2.31]
Random Effects 0.57 [0.33, 1.00]
I2= 0.00%
Q = 0.27; (p = 0.97)
Adjusted
Kinoshita, 2016 0.51 [0.20, 1.32]
0.05
0.15
1
2.5
5
10
30
Hazard Ratio (log scale)

B

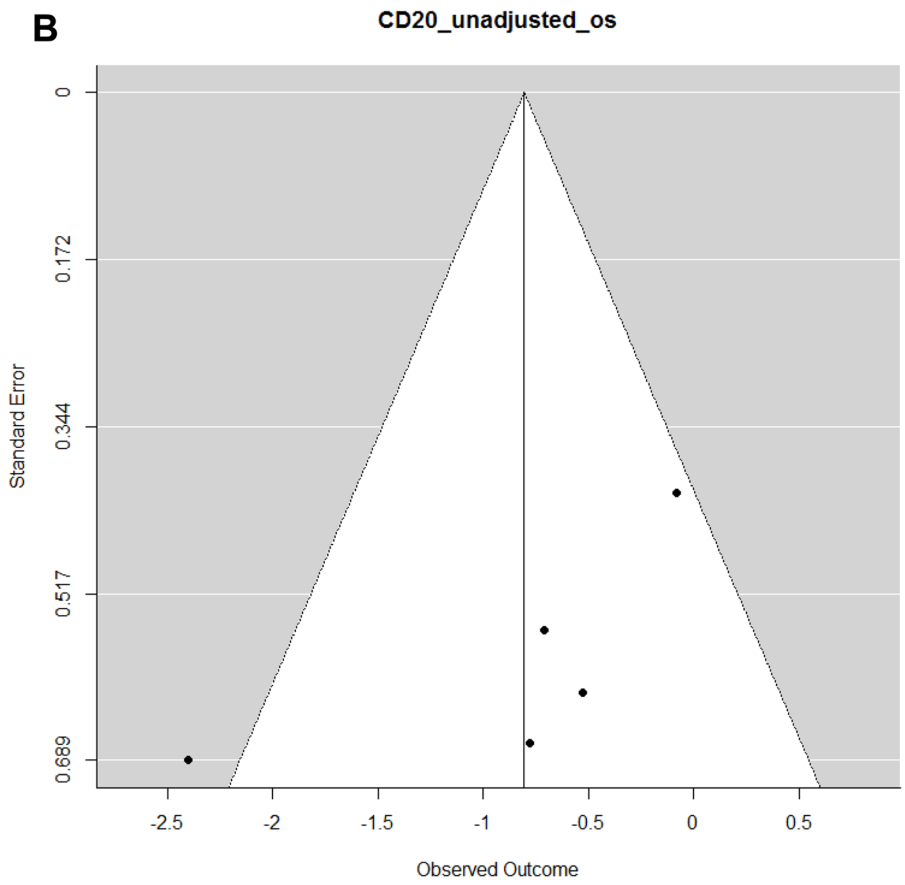


C

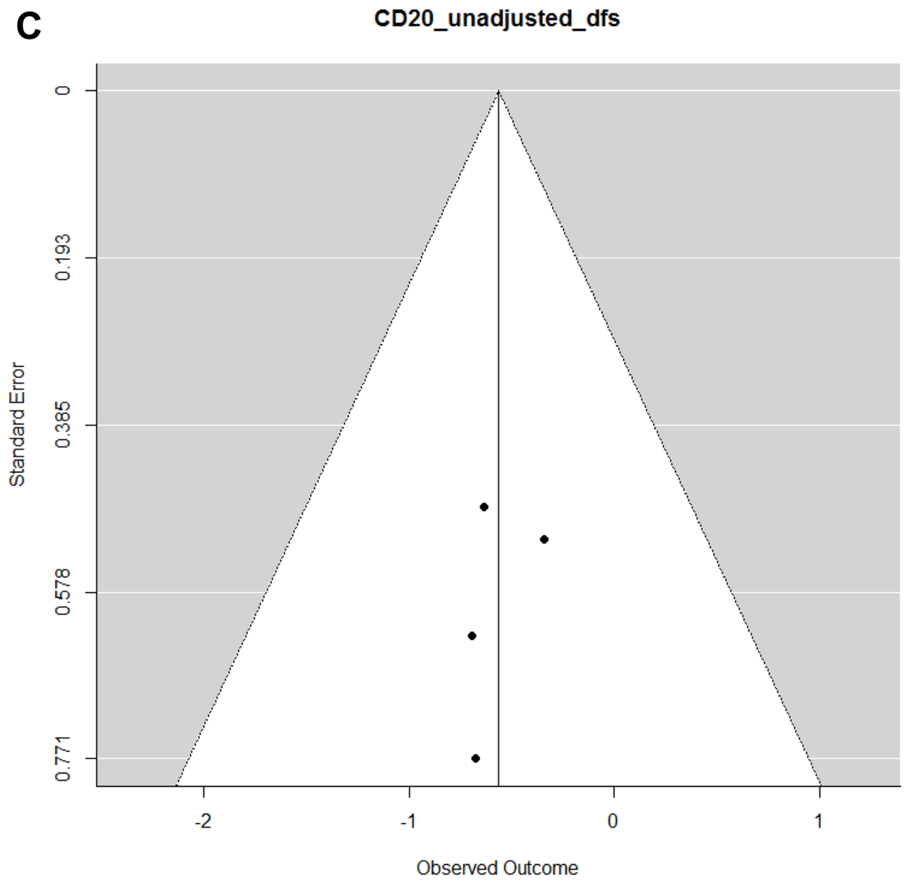


**Supplementary Figure 4:** (**A**) Forest Plot of CD20+ B cells. (**B**) Funnel Plot of CD20+ B cells Unadjusted OS Studies. (**C**) Funnel Plot of CD20+ B cells Unadjusted DFS Studies.

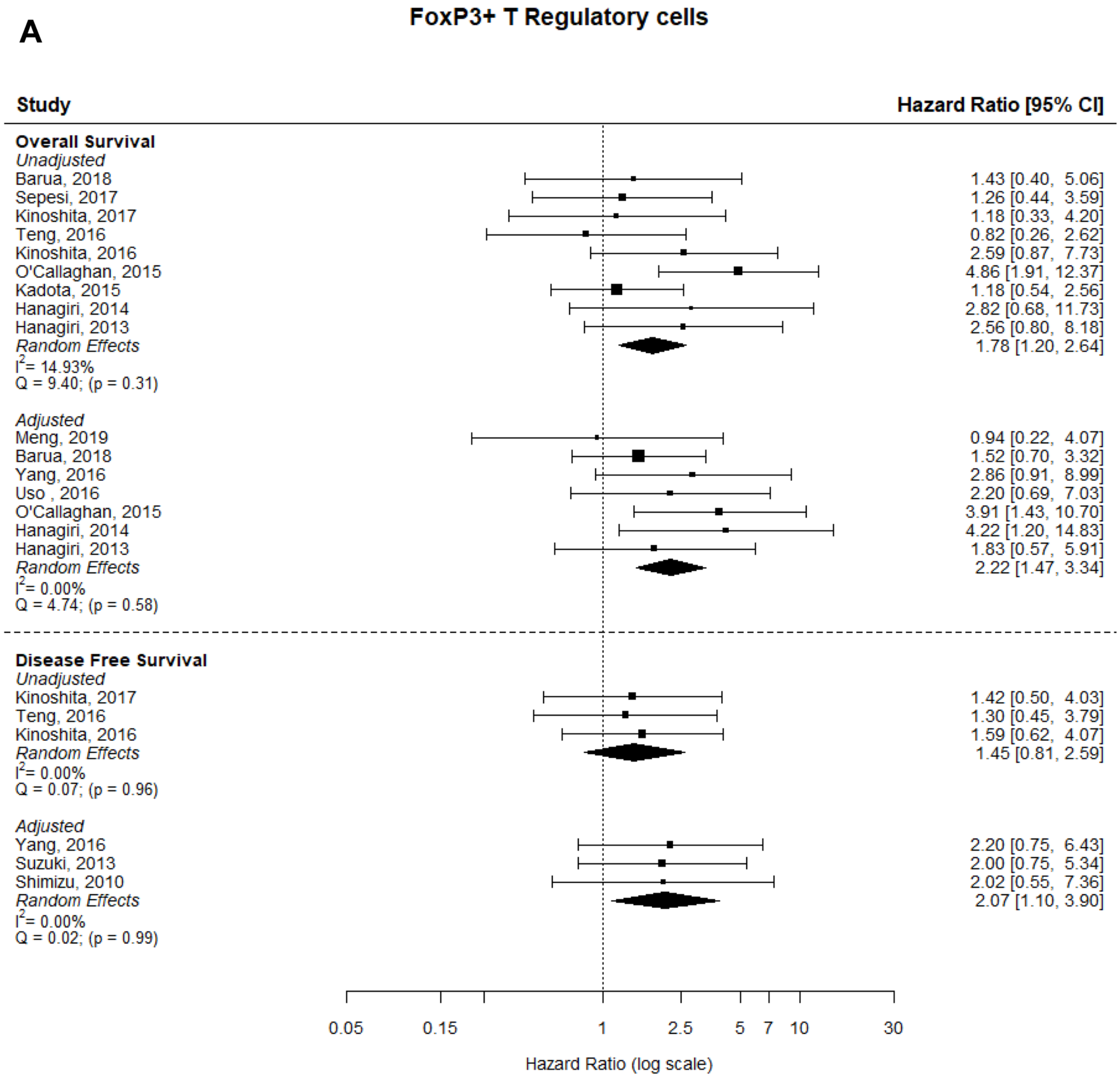
A
FoxP3+ T Regulatory cells
Study
Hazard Ratio [95% CI]
Overall Survival
Unadjusted
Barua, 2018 1.43 [0.40, 5.06]
Sepesi, 2017 1.26 [0.44, 3.59]
Kinoshita, 2017 1.18 [0.33, 4.20]
Teng, 2016 0.82 [0.26, 2.62]
Kinoshita, 2016 2.59 [0.87, 7.73]
O'Callaghan, 2015 4.86 [1.91, 12.37]
Kadota, 2015 1.18 [0.54, 2.56]
Hanagiri, 2014 2.82 [0.68, 11.73]
Hanagiri, 2013 2.56 [0.80, 8.18]
Random Effects 1.78 [1.20, 2.64]
I2= 14.93%
Q = 9.40; (p = 0.31)
Adjusted
Meng, 2019 0.94 [0.22, 4.07]
Barua, 2018 1.52 [0.70, 3.32]
Yang, 2016 2.86 [0.91, 8.99]
Uso , 2016 2.20 [0.69, 7.03]
O'Callaghan, 2015 3.91 [1.43, 10.70]
Hanagiri, 2014 4.22 [1.20, 14.83]
Hanagiri, 2013 1.83 [0.57, 5.91]
Random Effects 2.22 [1.47, 3.34]
I2= 0.00%
Q = 4.74; (p = 0.58)
Disease Free Survival
Unadjusted
Kinoshita, 2017 1.42 [0.50, 4.03]
Teng, 2016 1.30 [0.45, 3.79]
Kinoshita, 2016 1.59 [0.62, 4.07]
Random Effects 1.45 [0.81, 2.59]
I2= 0.00%
Q = 0.07; (p = 0.96)
Adjusted
Yang, 2016 2.20 [0.75, 6.43]
Suzuki, 2013 2.00 [0.75, 5.34]
Shimizu, 2010 2.02 [0.55, 7.36]
Random Effects 2.07 [1.10, 3.90]
I2= 0.00%
Q = 0.02; (p = 0.99)
0.05
0.15
1
2.5
5
7
10
30
Hazard Ratio (log scale)

B

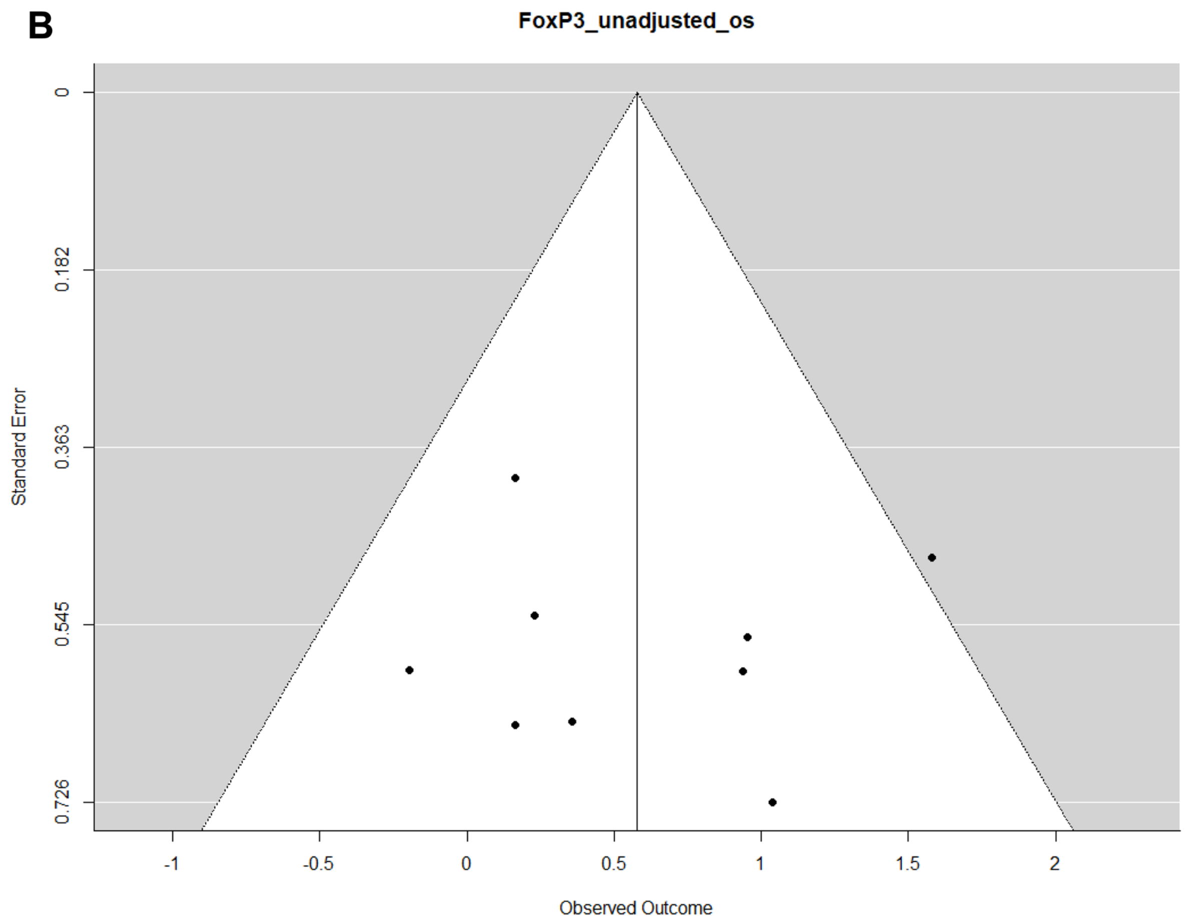
FoxP3_unadjusted_os
Standard Error
0
0.182
0.363
0.545
0.726
-1
-0.5
0
0.5
1
1.5
2
Observed Outcome

C

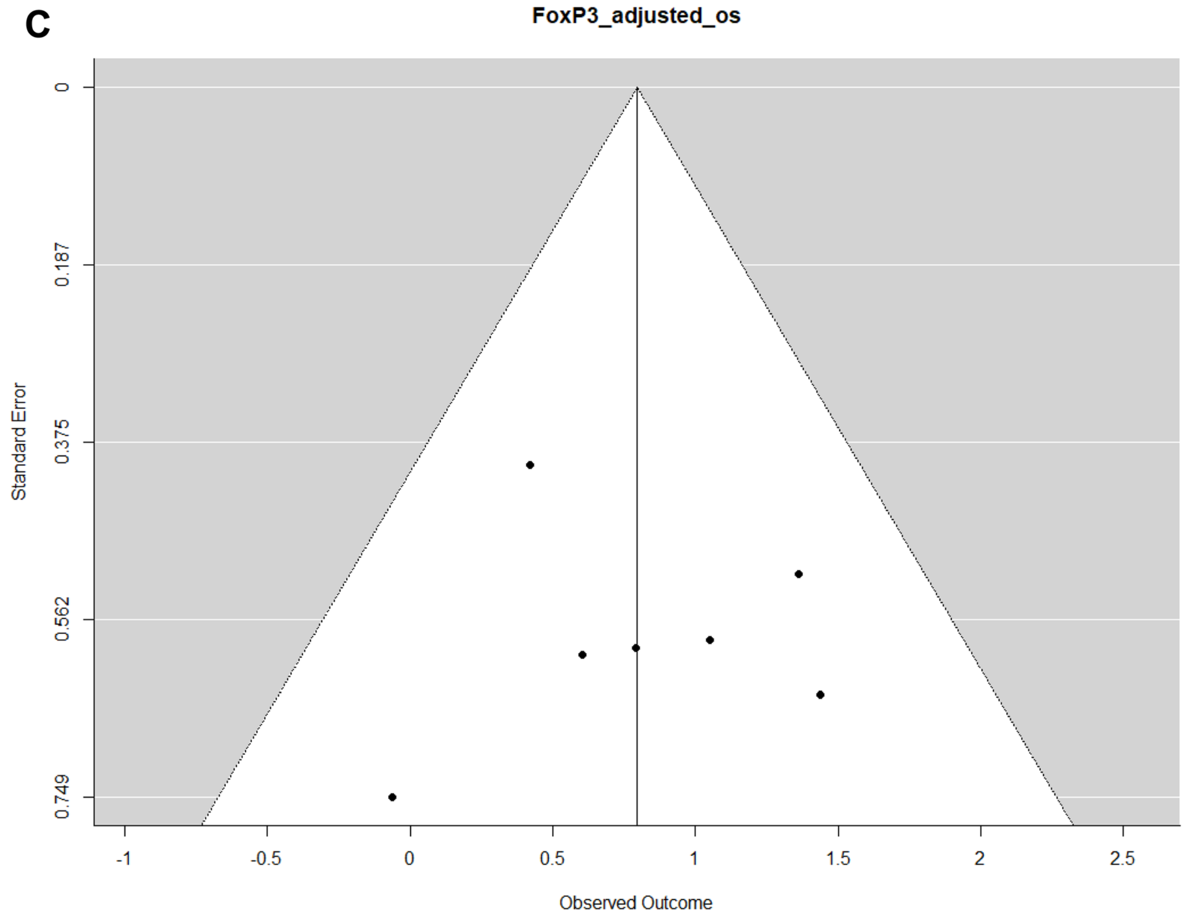
FoxP3_adjusted_os
Standard Error
0
0.187
0.375
0.562
0.749
-1
-0.5
0
0.5
1
1.5
2
2.5
Observed Outcome

D

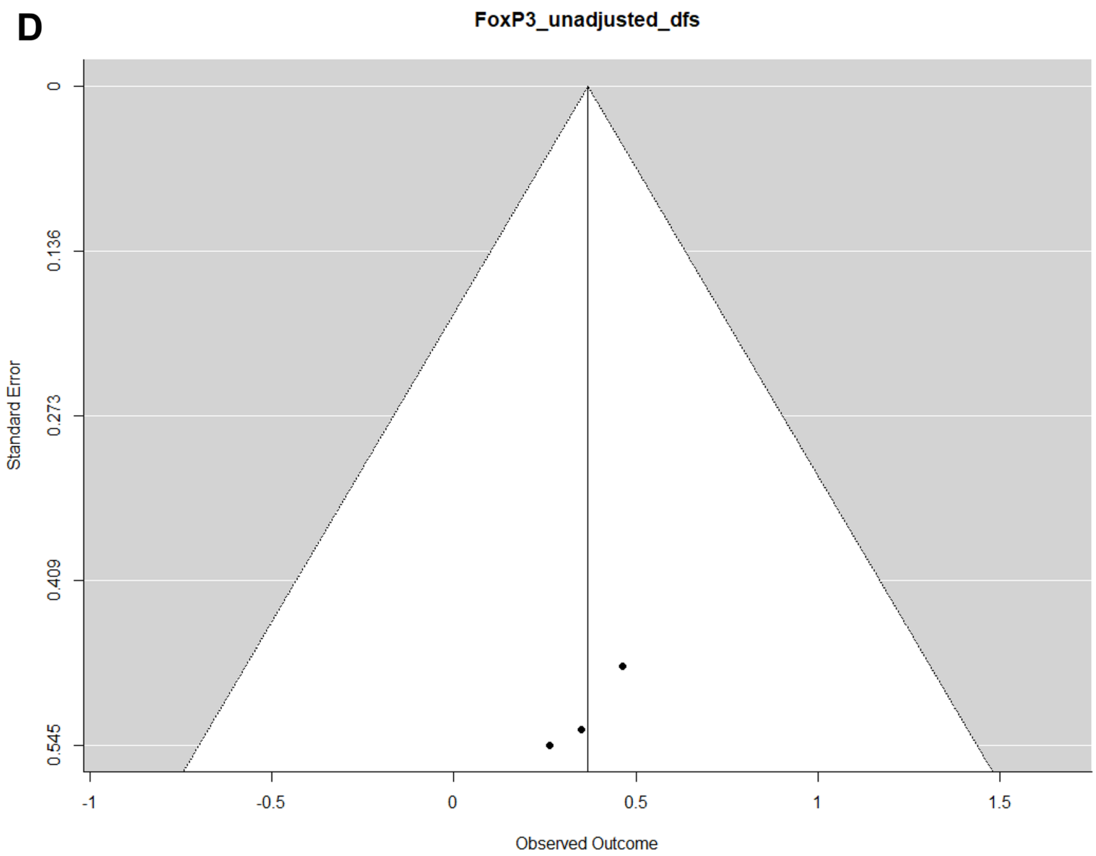


E

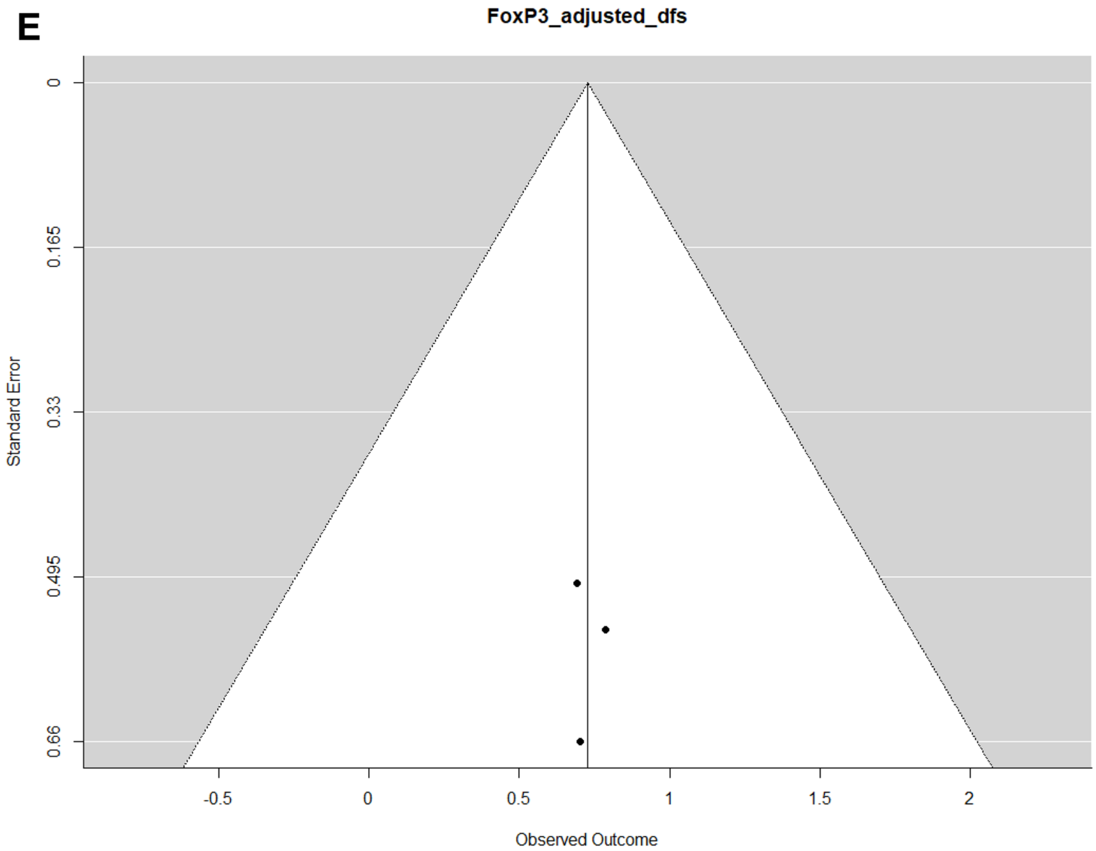


**Supplementary Figure 5:** (**A**) Forest Plot of FoxP3+T Regulatory cells. (**B**) Funnel Plot of FoxP3+T Regulatory cells Unadjusted OS Studies. (**C**) Funnel Plot of FoxP3+T Regulatory cells Adjusted OS Studies. (**D**) Funnel Plot of FoxP3+T Regulatory cells Unadjusted DFS Studies. (**E**) Funnel Plot of FoxP3+T Regulatory cells Adjusted DFS Studies.

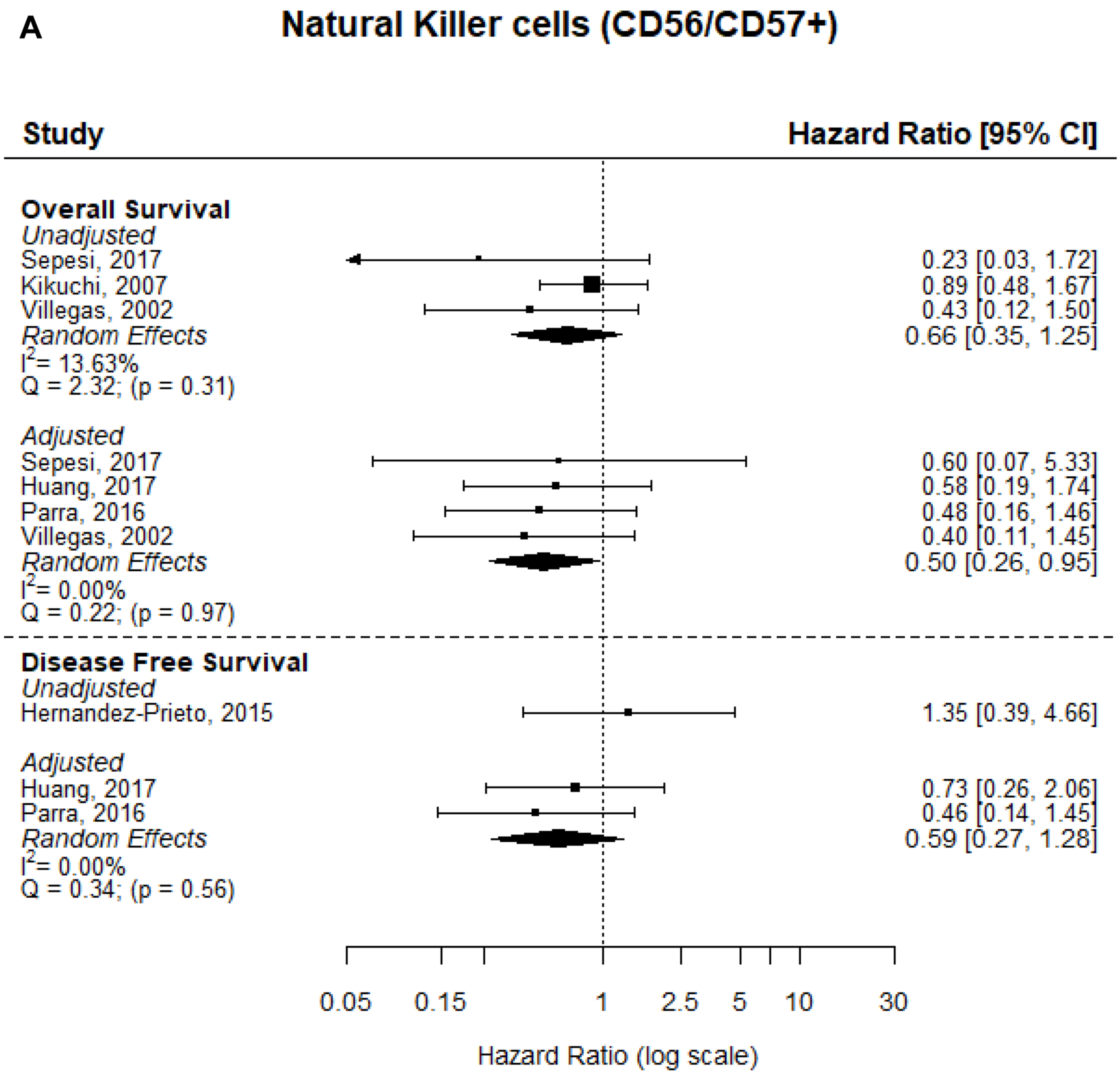
A
Natural Killer cells (CD56/CD57+)
Study
Hazard Ratio [95% CI]
Overall Survival
Unadjusted
Sepesi, 2017
Kikuchi, 2007
Villegas, 2002
Random Effects
I2= 13.63%
Q = 2.32; (p = 0.31)
0.23 [0.03, 1.72]
0.89 [0.48, 1.67]
0.43 [0.12, 1.50]
0.66 [0.35, 1.25]
Adjusted
Sepesi, 2017
Huang, 2017
Parra, 2016
Villegas, 2002
Random Effects
I2= 0.00%
Q = 0.22; (p = 0.97)
0.60 [0.07, 5.33]
0.58 [0.19, 1.74]
0.48 [0.16, 1.46]
0.40 [0.11, 1.45]
0.50 [0.26, 0.95]
Disease Free Survival
Unadjusted
Hernandez-Prieto, 2015
1.35 [0.39, 4.66]
Adjusted
Huang, 2017
Parra, 2016
Random Effects
I2= 0.00%
Q = 0.34; (p = 0.56)
0.73 [0.26, 2.06]
0.46 [0.14, 1.45]
0.59 [0.27, 1.28]
0.05
0.15
1
2.5
5
10
30
Hazard Ratio (log scale)

B

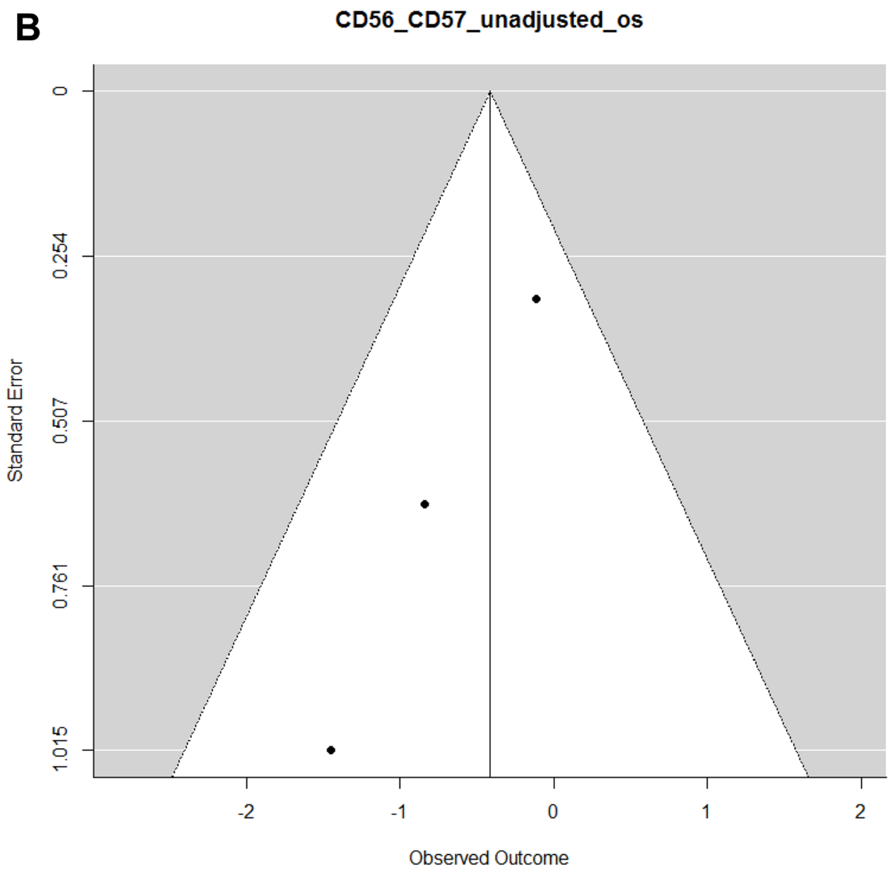


C

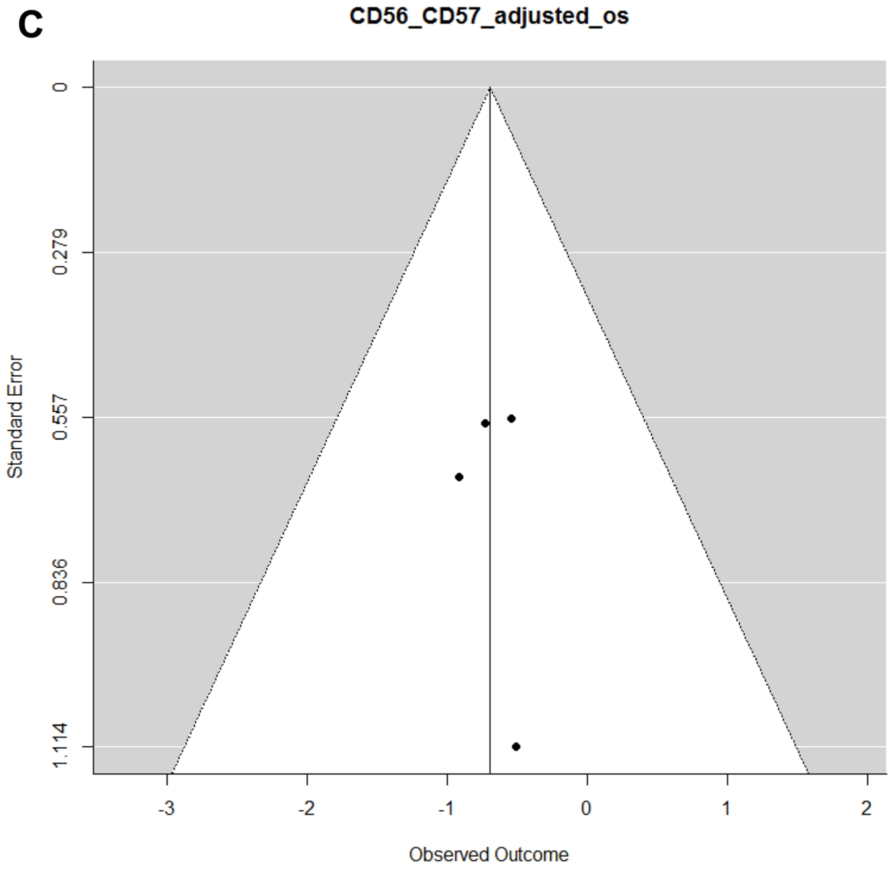


**Supplementary Figure 6:** (**A**) Forest Plot of NK cells (CD56/CD57+). (**B**) Funnel Plot of NK cells (CD56/CD57+) Unadjusted OS Studies. (**C**) Funnel Plot of NK cells (CD56/CD57+) Adjusted OS Studies.

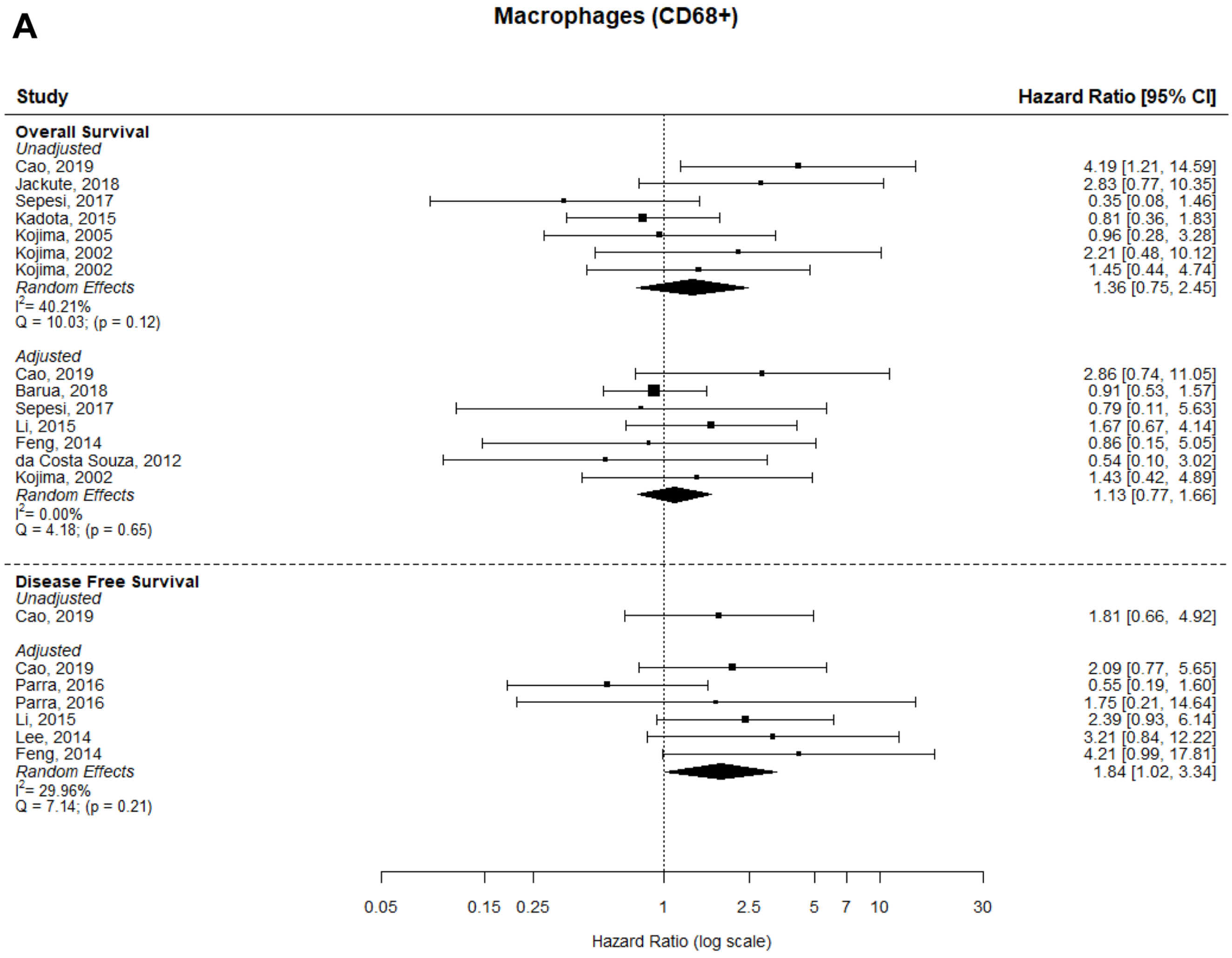
A
Macrophages (CD68+)
Study
Hazard Ratio [95% CI]
Overall Survival
Unadjusted
Cao, 2019 4.19 [1.21, 14.59]
Jackute, 2018 2.83 [0.77, 10.35]
Sepesi, 2017 0.35 [0.08, 1.46]
Kadota, 2015 0.81 [0.36, 1.83]
Kojima, 2005 0.96 [0.28, 3.28]
Kojima, 2002 2.21 [0.48, 10.12]
Kojima, 2002 1.45 [0.44, 4.74]
Random Effects 1.36 [0.75, 2.45]
$I^2$= 40.21%
Q = 10.03; (p = 0.12)
Adjusted
Cao, 2019 2.86 [0.74, 11.05]
Barua, 2018 0.91 [0.53, 1.57]
Sepesi, 2017 0.79 [0.11, 5.63]
Li, 2015 1.67 [0.67, 4.14]
Feng, 2014 0.86 [0.15, 5.05]
da Costa Souza, 2012 0.54 [0.10, 3.02]
Kojima, 2002 1.43 [0.42, 4.89]
Random Effects 1.13 [0.77, 1.66]
$I^2$= 0.00%
Q = 4.18; (p = 0.65)
Disease Free Survival
Unadjusted
Cao, 2019 1.81 [0.66, 4.92]
Adjusted
Cao, 2019 2.09 [0.77, 5.65]
Parra, 2016 0.55 [0.19, 1.60]
Parra, 2016 1.75 [0.21, 14.64]
Li, 2015 2.39 [0.93, 6.14]
Lee, 2014 3.21 [0.84, 12.22]
Feng, 2014 4.21 [0.99, 17.81]
Random Effects 1.84 [1.02, 3.34]
$I^2$= 29.96%
Q = 7.14; (p = 0.21)
0.05 0.15 0.25 1 2.5 5 7 10 30
Hazard Ratio (log scale)

B

CD68_unadjusted_os

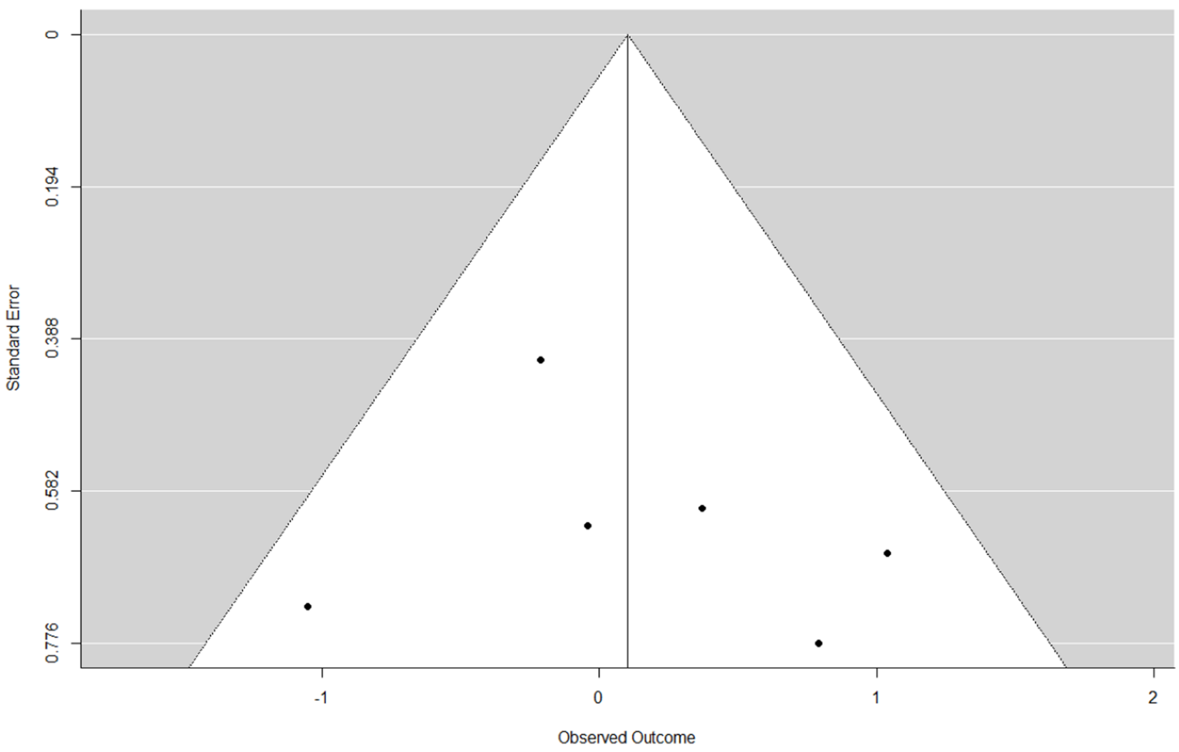


C

CD68_adjusted_os

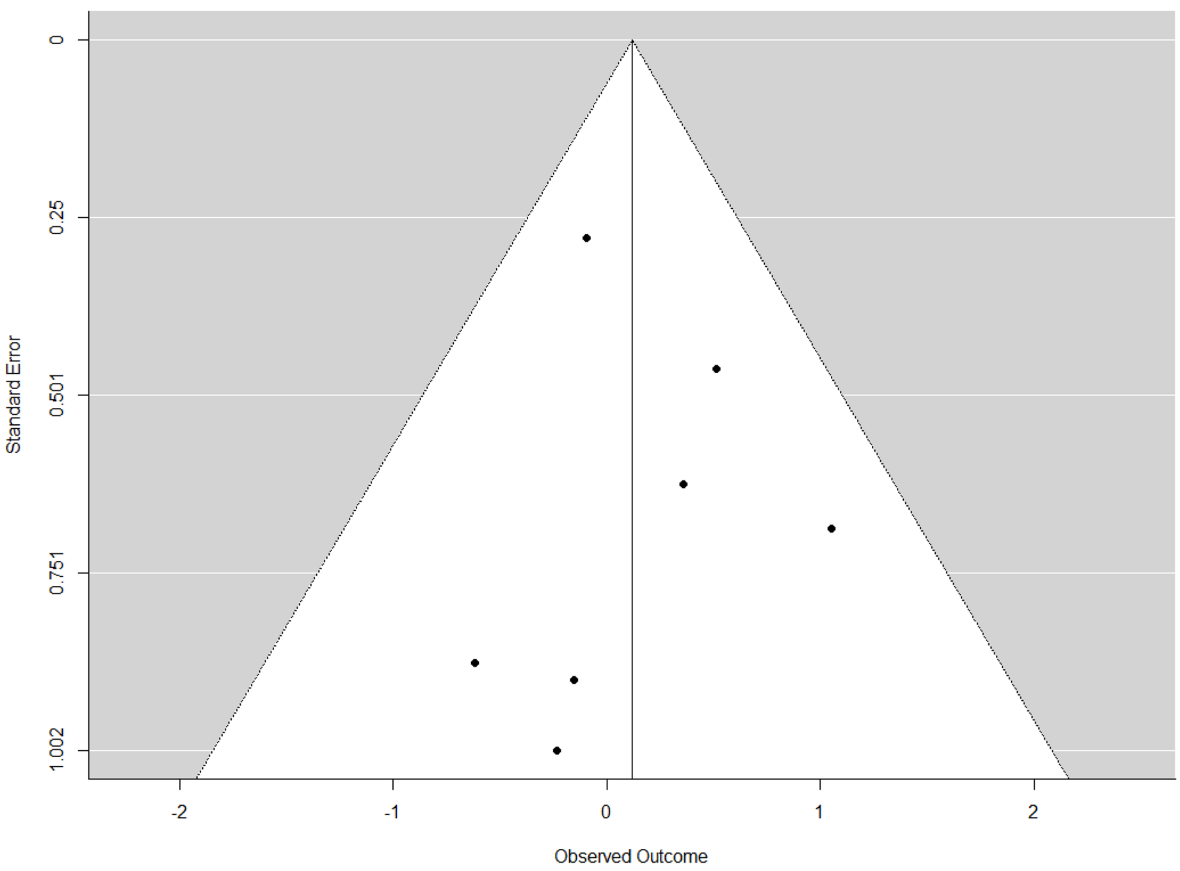

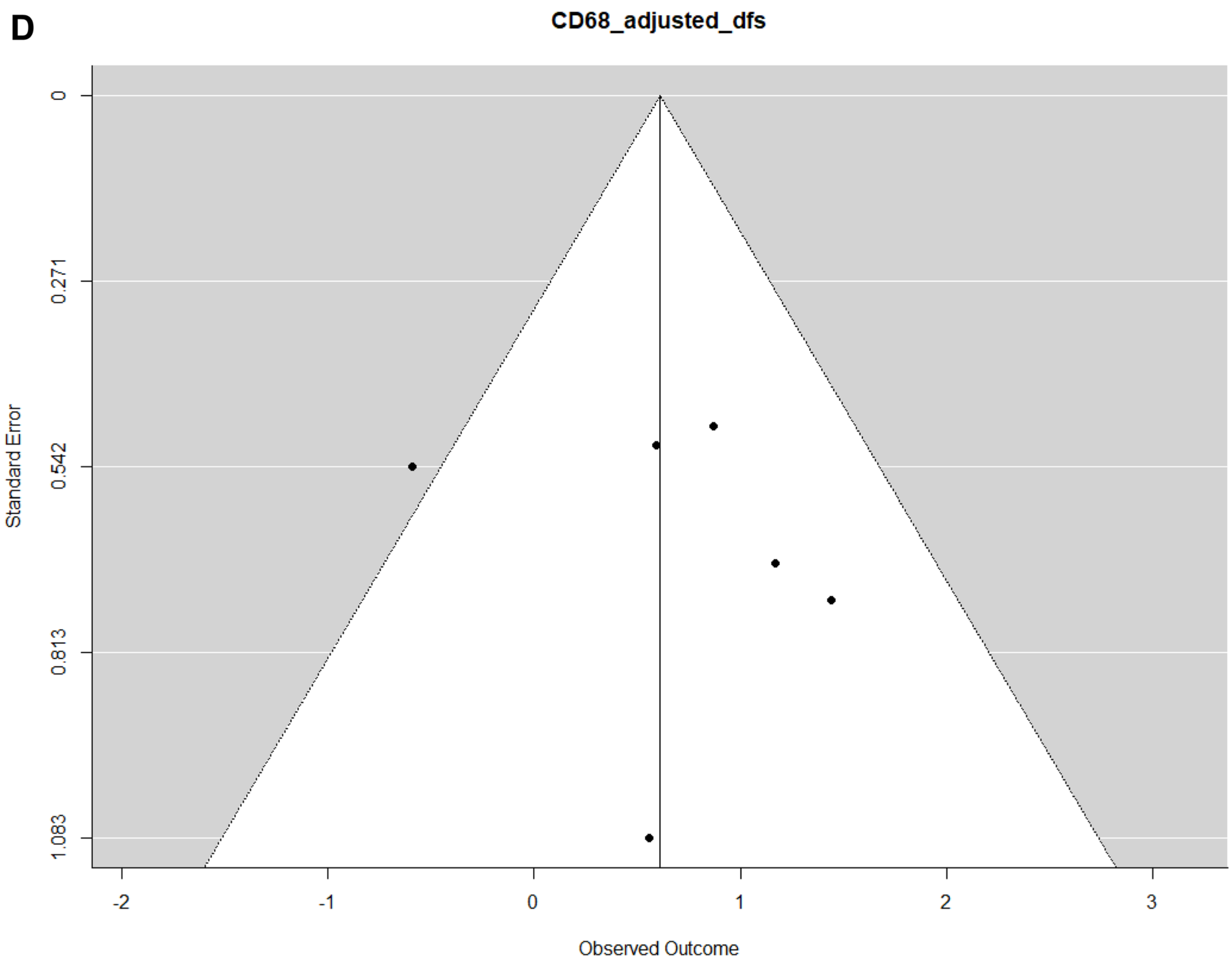


**Supplementary Figure 7:** (**A**) Forest Plot of macrophages (CD68+). (**B**) Funnel Plot of macrophages (CD68+) Unadjusted OS Studies. (**C**) Funnel Plot of macrophages (CD68+) Adjusted OS Studies. (**D**) Funnel Plot of macrophages (CD68+) Adjusted DFS Studies.

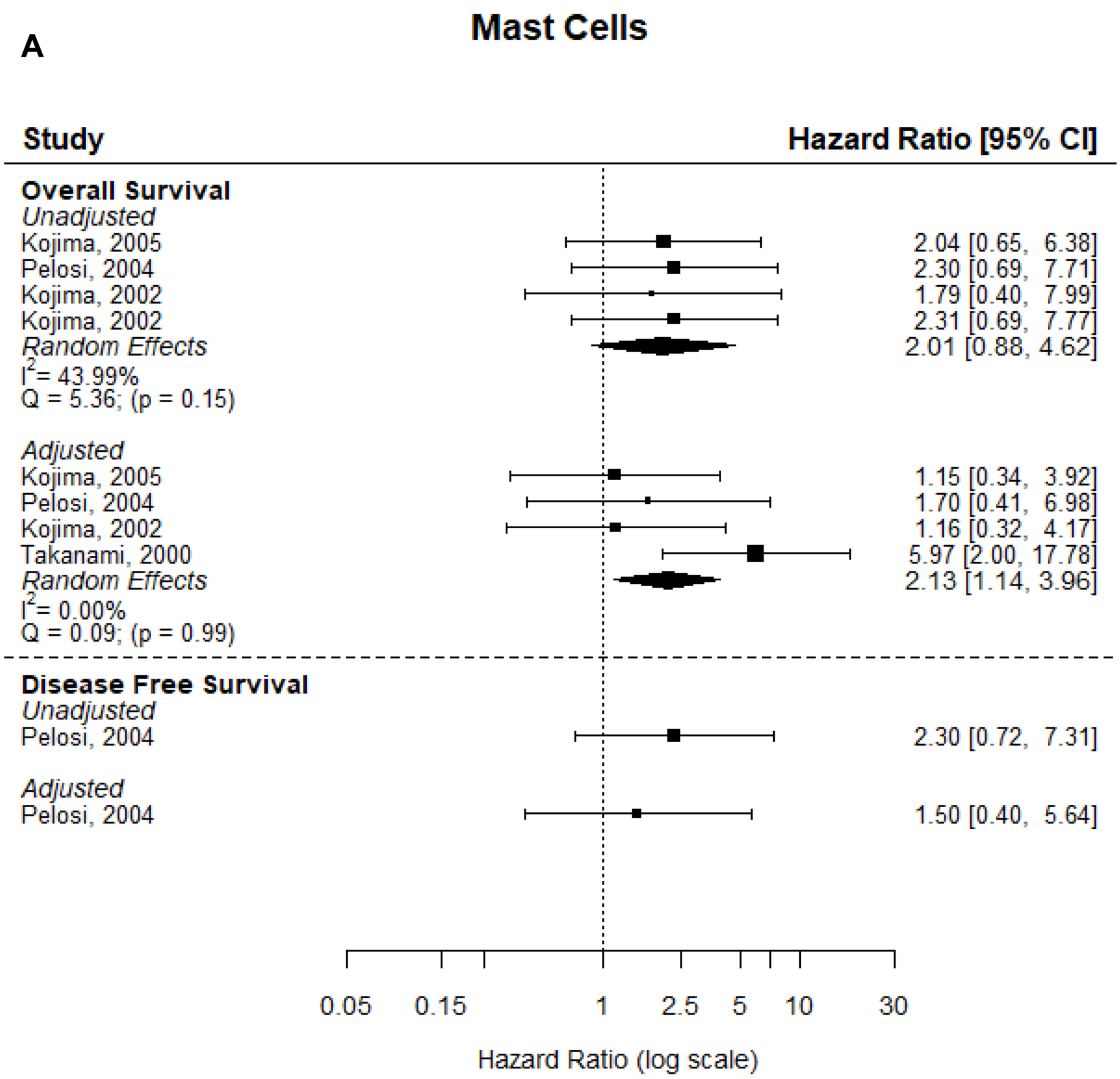
A
Mast Cells
Study
Hazard Ratio [95% CI]
Overall Survival
Unadjusted
Kojima, 2005 2.04 [0.65, 6.38]
Pelosi, 2004 2.30 [0.69, 7.71]
Kojima, 2002 1.79 [0.40, 7.99]
Kojima, 2002 2.31 [0.69, 7.77]
Random Effects 2.01 [0.88, 4.62]
I2= 43.99%
Q = 5.36; (p = 0.15)
Adjusted
Kojima, 2005 1.15 [0.34, 3.92]
Pelosi, 2004 1.70 [0.41, 6.98]
Kojima, 2002 1.16 [0.32, 4.17]
Takanami, 2000 5.97 [2.00, 17.78]
Random Effects 2.13 [1.14, 3.96]
I2= 0.00%
Q = 0.09; (p = 0.99)
Disease Free Survival
Unadjusted
Pelosi, 2004 2.30 [0.72, 7.31]
Adjusted
Pelosi, 2004 1.50 [0.40, 5.64]
0.05
0.15
1
2.5
5
10
30
Hazard Ratio (log scale)

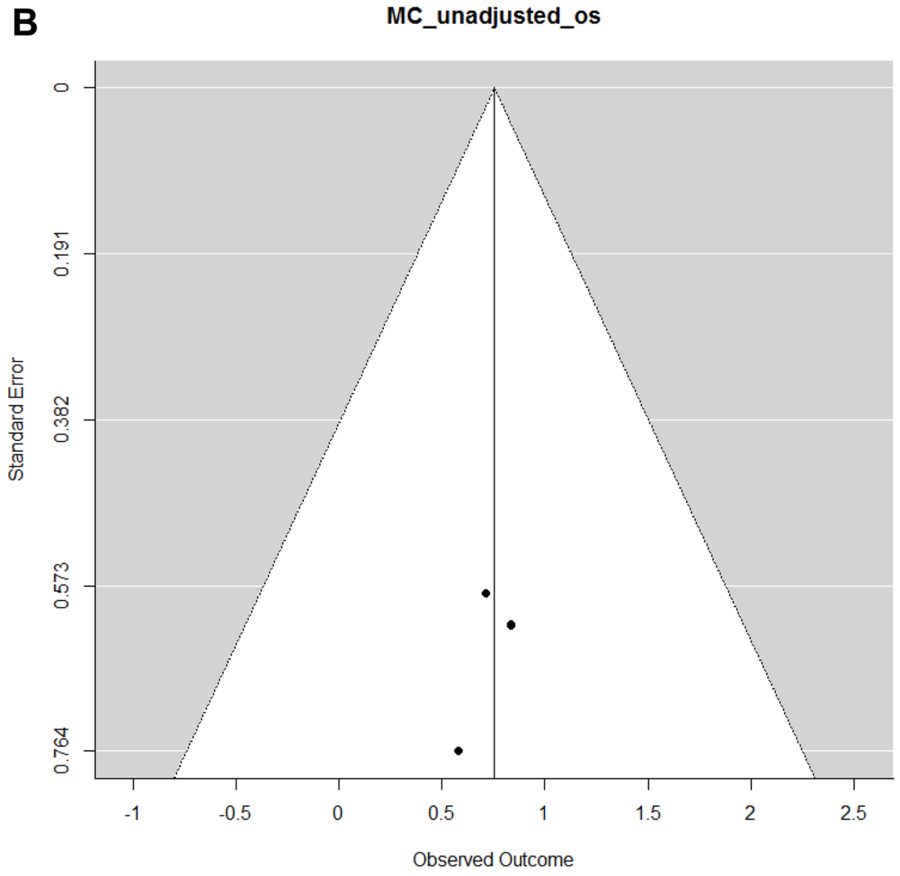


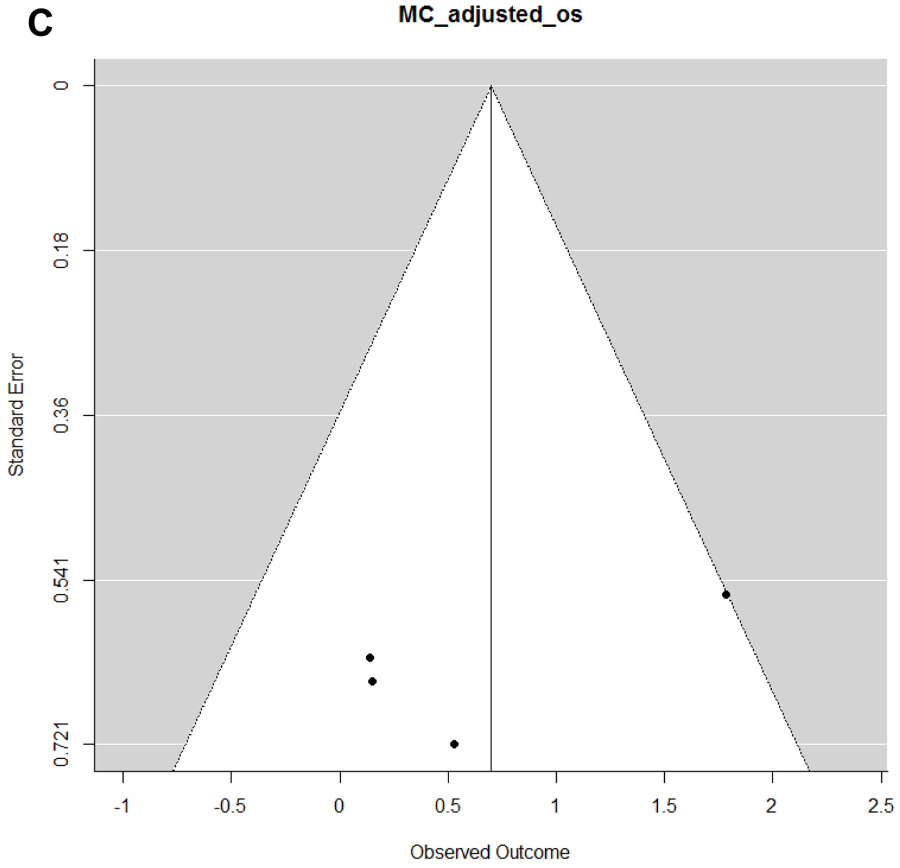


**Supplementary Figure 8:** (**A**) Forest Plot of MCs. (**B**) Funnel Plot of MCs Unadjusted OS Studies. (**C**) Funnel Plot of MCs Adjusted OS Studies.
